# Regulation of Pyridine Nucleotide Metabolism During Tomato Fruit Development Through Transcript and Protein Profiling

**DOI:** 10.3389/fpls.2019.01201

**Published:** 2019-10-11

**Authors:** Guillaume Decros, Bertrand Beauvoit, Sophie Colombié, Cécile Cabasson, Stéphane Bernillon, Stéphanie Arrivault, Manuela Guenther, Isma Belouah, Sylvain Prigent, Pierre Baldet, Yves Gibon, Pierre Pétriacq

**Affiliations:** ^1^UMR 1332 BFP, INRA, Univ. Bordeaux, Villenave d’Ornon, France; ^2^MetaboHUB-Bordeaux, MetaboHUB, Phenome-Emphasis, Villenave d’Ornon, France; ^3^Department 2, Metabolic Networks, Max Planck Institute of Molecular Plant Physiology, Potsdam-Golm, Germany

**Keywords:** fruit, tomato, NAD, redox, development

## Abstract

Central metabolism is the engine of plant biomass, supplying fruit growth with building blocks, energy, and biochemical cofactors. Among metabolic cornerstones, nicotinamide adenine dinucleotide (NAD) is particularly pivotal for electron transfer through reduction–oxidation (redox) reactions, thus participating in a myriad of biochemical processes. Besides redox functions, NAD is now assumed to act as an integral regulator of signaling cascades involved in growth and environmental responses. However, the regulation of NAD metabolism and signaling during fruit development remains poorly studied and understood. Here, we benefit from RNAseq and proteomic data obtained from nine growth stages of tomato fruit (var. Moneymaker) to dissect mRNA and protein profiles that link to NAD metabolism, including *de novo* biosynthesis, recycling, utilization, and putative transport. As expected for a cofactor synthesis pathway, protein profiles failed to detect enzymes involved in NAD synthesis or utilization, except for nicotinic acid phosphoribosyltransferase (NaPT) and nicotinamidase (NIC), which suggested that most NAD metabolic enzymes were poorly represented quantitatively. Further investigations on transcript data unveiled differential expression patterns during fruit development. Interestingly, among specific NAD metabolism-related genes, early *de novo* biosynthetic genes were transcriptionally induced in very young fruits, in association with NAD kinase, while later stages of fruit growth rather showed an accumulation of transcripts involved in later stages of *de novo* synthesis and in NAD recycling, which agreed with augmented NAD(P) levels. In addition, a more global overview of 119 mRNA and 78 protein significant markers for NAD(P)-dependent enzymes revealed differential patterns during tomato growth that evidenced clear regulations of primary metabolism, notably with respect to mitochondrial functions. Overall, we propose that NAD metabolism and signaling are very dynamic in the developing tomato fruit and that its differential regulation is certainly critical to fuel central metabolism linking to growth mechanisms.

## Introduction

Plant metabolism is maintained by universal metabolic cornerstones including pyridine nucleotides such as nicotinamide adenine dinucleotide (NAD) (Noctor et al., 2006; Gakière et al., 2018b). NAD and its phosphorylated form NADP are ubiquitous electron carriers modulating energy homeostasis through the transport of electrons within reduction–oxidation (redox) processes (Geigenberger and Fernie, 2014; Gakière et al., 2018a). As a result of its capacity to transfer electrons, NAD(P) is present in the cell as oxidized or reduced forms, NAD(P)^+^, and NAD(P)H, respectively, where NAD(P) refer to as the total pool of NAD(P)^+^ and NAD(P)H. In plant cells, while NAD is mostly found as oxidized NAD^+^, NADP mostly acts as a reductant (NADPH) (Noctor et al., 2006; Gakière et al., 2018b). For instance, the regeneration of reducing equivalents (NADPH) by the oxidative pentose phosphate pathway is necessary for the β-oxidation of fatty acids and for nitrogen assimilation in non-photosynthetic tissues (Neuhaus and Emes, 2000; Bowsher et al., 2007). Phosphorylation of NAD(H) to NADP(H) is catalyzed *via* highly conserved NAD^+^ kinases (E.C. 2.7.1.23) and NADH kinases (E.C. 2.7.1.86) playing essential roles in metabolic and redox reactions including photosynthesis performance and reactive oxygen species (ROS) homeostasis, which are both crucial for plant growth and responses to stress (Turner et al., 2004; Waller et al., 2010; Li et al., 2014; Li et al., 2018a).

**Figure 1:**
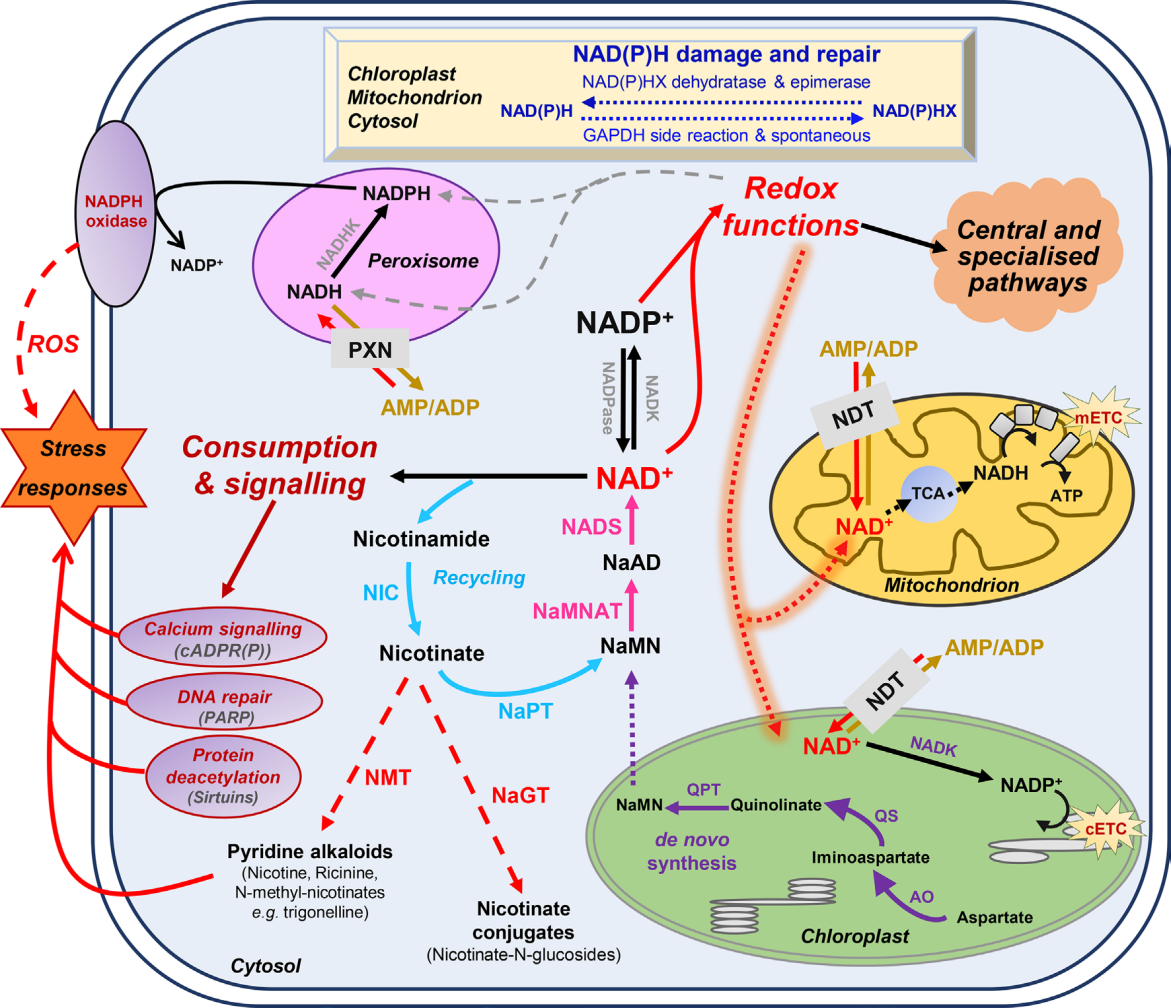
Basics of nicotinamide adenine dinucleotide (NAD^+^) metabolism in plant cells. Biosynthesis and utilization of pyridine nucleotides (Pétriacq et al., 2013). Dot arrows represent transport between the different cellular organelles. Purple and blue arrows indicate *de novo* synthesis pathway of NAD^+^, and the recycling pathway, respectively. Pink arrows represent steps that are shared by these two synthesis pathways. Dashed red arrows indicate nicotinate metabolism. Indigo arrows show NAD(P)H damage and repair, which can be spontaneous or catalyzed by NAD(P)H-hydrate dehydratase and epimerase. AO, aspartate oxidase; ATP, adenosine triphosphate; cADPR(P), cyclic ADP-ribose (phosphate); cETC, chloroplastic electron transport chain; GAPDH, glyceraldehyde-3-phosphate dehydrogenase; mETC, mitochondrial electron transport chain; NaAD, nicotinic acid adenine dinucleotide; NADK, NAD kinase; NADP, NAD phosphate; NADPase, NADP phosphatase; NAD(P)HX, NAD(P)H hydrate; NaMN, nicotinic acid mononucleotide; NaMNAT, NaMN adenylyltransferase; NADS, NAD synthetase; NaGT, nicotinate N-glucosyltransferase; NaPT, nicotinate phosphoribosyltransferase; NDT, NAD^+^ transporter; NIC, nicotinamidase; NMT, nicotinate N-methyltransferase; PARP, poly-ADP-ribose polymerase; PXN, peroxisomal NAD carrier; QPT, quinolinate phosphoribosyltransferase; QS, quinolinate synthase; ROS, reactive oxygen species; TCA, tricarboxylic acid cycle.

Plant cells produce NAD^+^ from the amino acid aspartate *via* a *de novo* biosynthesis and a recycling pathway (**Figure 1**) (Katoh et al., 2006; Gakière et al., 2018b). Briefly, *de novo* synthesis starts with quinolinate formation in the chloroplast (Katoh et al., 2006) from aspartate and dihydroxyacetone phosphate by aspartate oxidase (AO; E.C. 1.4.3.16) plus quinolinate synthase (QS; E.C. 2.5.1.72) (**Figure 1**). Quinolinate phosphoribosyltransferase (QPT; E.C. 2.4.2.19) catalyzes the subsequent conversion of quinolinate to nicotinic acid (Na) mononucleotide (NaMN). The next biochemical reactions are cytosolic (Suda et al., 2003; Magni et al., 2004; Noctor et al., 2006; Hashida et al., 2007) and shared between NAD biosynthesis and recycling. This thus requires the transfer of NaMN to the cytosol, the transporter for which remains to be discovered. NaMN is adenylylated to nicotinic acid adenine dinucleotide (NaAD) by nicotinate mononucleotide adenylyl transferase (NaMNAT; E.C. 2.7.7.1); then, NAD synthetase (NADS; E.C. 6.3.1.5) finally amidates NaAD to NAD^+^ (Gakière et al., 2018b). As for other nucleotides that are salvaged, NAD^+^ can be recycled *via* the activity of nicotinamidases (NIC; E.C. 3.5.1.19) and nicotinic acid phosphorybosyltransferase (NaPT; E.C. 2.4.2.11) (**Figure 1**). Due to its toxicity, nicotinate is stored in plant cells as Na-conjugates such as nicotinate glucosides and trigonelline (*i.e.*, N-methylnicotinate) by glycosylation *via* Na glucosyltransferase (NaGT; E.C. 2.4.1.196) or methylation *via* Na-N-methyltransferase (NMT; E.C. 2.1.1.7) (Köster et al., 1989; Li et al., 2015; Li et al., 2017). While the topology of NAD^+^ synthesis is well documented, the molecular and regulatory details of the corresponding biochemical pathways remain largely unknown, especially for fruit tissues. Deregulation of AO, QS, and QPT levels in *Arabidopsis thaliana* leaves has shown that such enzymes are critical in the control of pyridine nucleotide levels and its derivatives (Schippers et al., 2008; Macho et al., 2012; Pétriacq et al., 2012; Pétriacq et al., 2016). Na-glucosides have been shown to play a role in seed germination and contribute to resynthesis of NAD^+^ in *Brassicaceae* (Li et al., 2017). Although the physiological role of trigonelline in plants remain unclear, trigonelline can also contribute, to a lesser extent, to the resynthesis of NAD^+^ and undergoes long-distance transport in *Arabidopsis* (Upmeier et al., 1988; Wu et al., 2018).

Besides their redox functions, pyridine nucleotides can be directly consumed by signaling reactions that influence plant physiology through developmental processes and responses to environmental changes, most particularly stress mitigations (Hashida et al., 2009; Pétriacq et al., 2013; Gakière et al., 2018a). This includes calcium signaling (**Figure 1**), translational modification of target proteins by ADP-ribose transfer from NAD^+^ *via* the poly-ADP-ribose polymerases (PARPs, E.C. 2.4.2.30) and mono-ADP-ribosyltransferase (E.C. 2.4.2.31), and epigenetic regulations *via* the sirtuin histone deacetylases (SIRs, E.C. 2.4.2.B14) (Hunt et al., 2004; Briggs and Bent, 2011; Kupis et al., 2016; Gakière et al., 2018b). Furthermore, Nudix hydrolases (NDXs, E.C. 2.4.2.13) can hydrolyze pyridine nucleotides and participate in signaling functions (Kraszewska, 2008). NAD(P)H can also undergo spontaneous and enzymatic hydrations that generate NAD(P)H hydrates, which can be subsequently repaired to NAD(P)H by NAD(P)H-hydrate dehydratases (E.C. 4.2.1.93/136) and epimerases (E.C. 5.1.99.6) (Niehaus et al., 2014). Several reviews have previously covered these aspects that link NAD^+^ signaling to plant development and stress responses (Mahalingam et al., 2007; Hashida et al., 2009; Pétriacq et al., 2013; Gakière et al., 2018a; Gakière et al., 2018b). For instance, research on pyridine nucleotide signaling mostly focuses on photosynthetic tissues, sometimes roots and seeds (Hunt et al., 2007; Hunt and Gray, 2009; Hashida et al., 2012). In *Arabidopsis*, changes in NAD(P)^+^ contents drastically alter growth phenotype, as exemplified particularly for genotypes that are affected for the first three enzymes of *de novo* biosynthesis (for a review see Gakière et al., 2018a). Following NAD(H) and NADP(H) levels in *Arabidopsis* developing leaves further indicate a continuous accumulation of the cofactors during foliar growth until the pools drop with flowering (Queval and Noctor, 2007). A positive correlation is observed between levels of NAD^+^ and the resistance of *Arabidopsis* plants to several biotic changes, including fungal and bacterial infections (Zhang and Mou, 2009; Macho et al., 2012; Pétriacq et al., 2012; Pétriacq et al., 2016). In citrus plants, recent work highlights the importance of exogenous NAD^+^ treatment in inducing resistance against citrus canker (Alferez et al., 2018). Furthermore, NAD^+^ might leak from the cellular compartment and subsequently induce immune responses, which is further supported by the discovery of a lectin receptor kinase as a potential receptor for NAD^+^ in *Arabidopsis*, also participating in basal resistance against bacterial pathogens (Zhang and Mou, 2009). Hence, the implication of pyridine nucleotides as signaling molecules is clearly established (Mou, 2017).

Only a handful of studies provide fruit-specific concentrations of NAD(H) and NAD(P)H. Although previous changes in pyridine nucleotides have been observed between green and red tomato fruits of MicroTom and Moneymaker cultivars (Centeno et al., 2011; Osorio et al., 2013), to our knowledge, no developmental fruit series have been analyzed so far. Tomato fruit is not only an important crop that is widely used for human diet but also pivotal for fruit research (Li et al., 2018b). During tomato fruit development, a medium-scale stoichiometric model suggested that biomass synthesis required NADPH and higher ATP hydrolysis at the end of cell expansion (Colombié et al., 2015). This was further associated with a peak of CO_2_ at the end of tomato ripening coinciding with climacteric respiration of tomato fruit and involved energy dissipation by the mitochondrial alternative oxidase. This was further confirmed by a more detailed stoichiometric model of the respiratory pathway, including alternative oxidase and uncoupling proteins (Colombié et al., 2017). In grape berry, reducing power equivalents (NADH and NADPH) were also associated with major carbon fluxes, thus supporting a strong link between central metabolism and pyridine nucleotides (Soubeyrand et al., 2018).

In the present work, as a first attempt to clarify the importance of pyridine nucleotides in the developing fruit, we used a developmental fruit series of nine growth stages of tomato fruit (var. Moneymaker) and measured pyridine nucleotide pools. We further examined quantitative data for transcript and protein levels previously obtained by RNAseq and proteomics (Belouah et al., 2019) and revealed changes in NAD(P) metabolism during fruit development. Our studies show that NAD(P) metabolism and signaling are very dynamic and crucial to the developing tomato fruit and link to central metabolism.

## Materials and Methods

### Plant Material and Growth Conditions

Tomato fruit pericarps were obtained from *Solanum lycopersicum* L. var. Moneymaker as previously described (Biais et al., 2014). Briefly, tomato plants were grown under usual production greenhouse conditions in southern western France (44°23ʹ56ʹʹN, 0°35ʹ25ʹʹE) from June to October, using a nutrient solution (detailed in Biais et al., 2014) to adjust plant growth and water supply to the climate *via* a drip irrigation system that maintained 20–30% drainage (pH adjusted to 5.9, electrical conductivity to 2.2 mS.cm^−1^). Flower anthesis was monitored on trusses 5, 6, and 7, and fruits were harvested at nine developmental stages (thereafter referred to as GS1 to 9), at about 8, 15, 21, 28, 34, 42 (mature green), 49 (turning), 50 (orange), and 53 (red ripe) days post anthesis (dpa). Seeds, jelly, and placenta were first removed for each fruit, and the resulting pericarp was cut into small pieces, which were immediately frozen in liquid nitrogen. Pericarp samples were ground to powder and stored at −80°C until further analysis.

### Quantification of NAD(P) Contents in Tomato Fruit

Total cellular soluble pools of NAD^+^, NADH, NADP^+^, and NADPH were measured from fruit pericarps of nine developmental stages of tomato fruit according to a coupled enzyme assay described previously (Pétriacq et al., 2012) using HiT-Me Facility at MetaboHUB-Bordeaux (https://doi.org/10.15454/1.5572412770331912E12 Plateforme Metabolome Bordeaux, http://metabolome.cgfb.u-bordeaux.fr/en). Briefly, from the same fresh ground fruit material (20 mg), oxidized forms NAD^+^ and NADP^+^ were extracted in 200 µl HCl (0.2 N) and reduced forms NADH and NADPH in 200 µl NaOH (0.2 N) as detailed in (Queval and Noctor, 2007). Microplate measurements of oxidized NAD^+^ and NADP^+^ (Pétriacq et al., 2012) were confirmed from methanolic extracts using ion-pair liquid chromatography coupled to mass spectrometry (LCMS) technique described previously (Arrivault et al., 2009). Resulting metabolite levels were expressed in nmol.g^−1^ fresh weight (FW) for independent bioreplicates (*n* = 3) and checked for statistical significance by ANOVA for global variation and by binary comparison of Student’s *t* test (*P* < 0.05). Volumes of cytosol and all organelles, except vacuole, were used to express metabolite pools as cellular concentrations by dividing pyridine nucleotide pools by the volume corresponding to each growth stage, as previously described (Beauvoit et al., 2014).

### RNAseq and LC-MS/MS Proteomics of Developing Tomato Fruit

RNA and proteins were extracted and analyzed as described previously (Belouah et al., 2019) by RNAseq (GeT-PlaGe core facility, INRA Toulouse, France, http://get.genotoul.fr) and LCMS/MS-based proteomics (PAPPSO, INRA Moulon, France, http://pappso.inra.fr/index.php), respectively. Briefly, total RNA was isolated from frozen tissue powder of tomato pericarp using plant RNA Reagent (PureLink Kit, Invitrogen) followed by DNase treatment (DNA-free Kit, Invitrogen) and purification over RNeasy Mini spin columns (RNeasy Plant Mini Kit, QIAGEN), according to the manufacturer’s instruction. RNA integrity was assessed using the RNA 600 Nano Kit with a Bioanalyzer 2100 system (Agilent Technologies). RNAseq was performed at the GeT-PlaGe core facility, INRA Toulouse (France). RNAseq libraries were prepared according to Illumina’s protocols using the TruSeq Stranded mRNA Sample Prep Kit to analyze mRNA. Library quality was assessed using an Agilent Bioanalyzer, and libraries were quantified by quantitative PCR (qPCR) using the Kapa Library Quantification Kit. RNAseq experiments were performed on an Illumina HiSeq 2000 or HiSeq 2500 (2x100 bp).

### Protein Extraction and Quantification

Total proteins from tomato pericarp were extracted as in (Faurobert et al., 2007). LC-MS/MS analyses were performed with a NanoLC-Ultra System (Nano-2D Ultra, Eksigent, Les Ulis, France) coupled with a Q Exactive Mass Spectrometer (Thermo Electron, Waltham, MA, USA) as in (Havé et al., 2018). For each sample, 800 ng (4 μl from a 0.200 ng.μl^−1^ solution) of protein digest were loaded onto a Biosphere C18 pre-column (0.1 × 20 mm, 100 Å, 5 μm; Nanoseparation) at 7.5 μl.min^−1^ and desalted with 0.1% (v/v) formic acid and 2% ACN. After 3 min, the pre-column was connected to a Biosphere C18 nanocolumn (0.075 × 300 mm, 100 Å, 3 μm; Nanoseparation). The raw MS output files and identification data were deposited online using the PROTICdb database (http://moulon.inra.fr/protic/tomato_fruit_development).

Protein identification was performed using the protein sequence database of *S. lycopersicum* Heinz assembly v 2.40 (ITAG2.4) downloaded from https://solgenomics.net/ (34,725 entries). A contaminant database, which contains the sequences of standard contaminants, was also interrogated. Criteria used for protein identification were (1) at least two different peptides identified with an E-value smaller than 0.01, and (2) a protein E-value (product of unique peptide E-values) smaller than 10^−5^. Using reversed sequences as a decoy database, the false discovery rate for peptide and protein identification was respectively 0.05 and 0%.

### Data Analysis of mRNA and Protein Profiles

Datasets consisted of 22,877 transcript and 2,375 protein profiles and were made publicly available *via* GEO repository (Barrett et al., 2013) with the accession number GSE12873 (https://www.ncbi.nlm.nih.gov/geo/query/acc.cgi?acc=GSE128739) for the transcripts. The proteomics data have been deposited to the ProteomeXchange Consortium *via* the PRIDE (Perez-Riverol et al., 2019) partner repository with the dataset identifier PXD012877.

Prior to uni- and multivariate statistical analyses, mRNA and protein data were pre-processed to normally distributed data by performing median normalization, cube-root transformation, and Pareto scaling of the data intensities as described previously (Belouah et al., 2019). Normalized datasets were then used to construct score plots of principal component analysis (PCA) for transcriptomic and proteomic overview using MetaboAnalyst v 4.0 (http://www.metaboanalyst.ca/), or dendrograms of clustering analysis by Pearson’s correlation with complete clustering linkage using MeV v 4.9.0 (http://mev.tm4.org/). Significant markers of both transcripts and proteins were determined by ANOVA after discarding false positives (*P* < 0.01 corrected for multiple testing by Bonferroni method). Details of ANOVA *P* values are given in **Supplemental Tables S1-3**. Transcript and protein features of NAD-dependent functions were selected by identifying domains (InterPro and GO) that were annotated to bind or process pyridine nucleotides using Assembly v 2.40 (ITAG v 2.4) from Sol Genomics (https://solgenomics.net/) and UniProt (https://www.uniprot.org/). Data mining of publicly available gene expression data was conducted using Tomato Expression Atlas (http://tea.solgenomics.net/) (Fernandez-Pozo et al., 2017). Functional annotation of mRNA and protein markers was performed based on gene ontology using Mercator4 v1.0 (https://plabipd.de/portal/mercator4) (Schwacke et al., 2019).

### Enzymatic Activities of Dehydrogenases

Malate dehydrogenase and isocitrate dehydrogenase activities were measured according to an in-house protocol (Biais et al., 2014). Briefly, aliquots of about 20-mg FW were extracted by vigorous shaking with 500 µl extraction buffer composed of 20% (v/v) glycerol, 0.25% (w/v) bovine serum albumin, 1% (v/v) Triton-X100, 50 mM HEPES-KOH (pH 7.5), 10 mM MgCl_2_, 1 mM EDTA, 1 mM EGTA, 1 mM ε-aminocaproic acid, 1 mM benzamidine, 10 mM leupeptin, 0.5 mM dithiothreitol, and 1 mM phenylmethylsulfonyl fluoride, which was added just before extraction. Enzyme activities were assayed using a robotized platform at HiT-Me Facility [(MetaboHUB-Bordeaux) http://metabolome.cgfb.u-bordeaux.fr/en] as previously described (Gibon et al., 2004; Studart-Guimarães et al., 2005; Gibon et al., 2006; Gibon et al., 2009; Steinhauser et al., 2010).

## Results

### Tomato Fruit Development Is Associated With Changes in NAD(P) Pools

**Figure 2:**
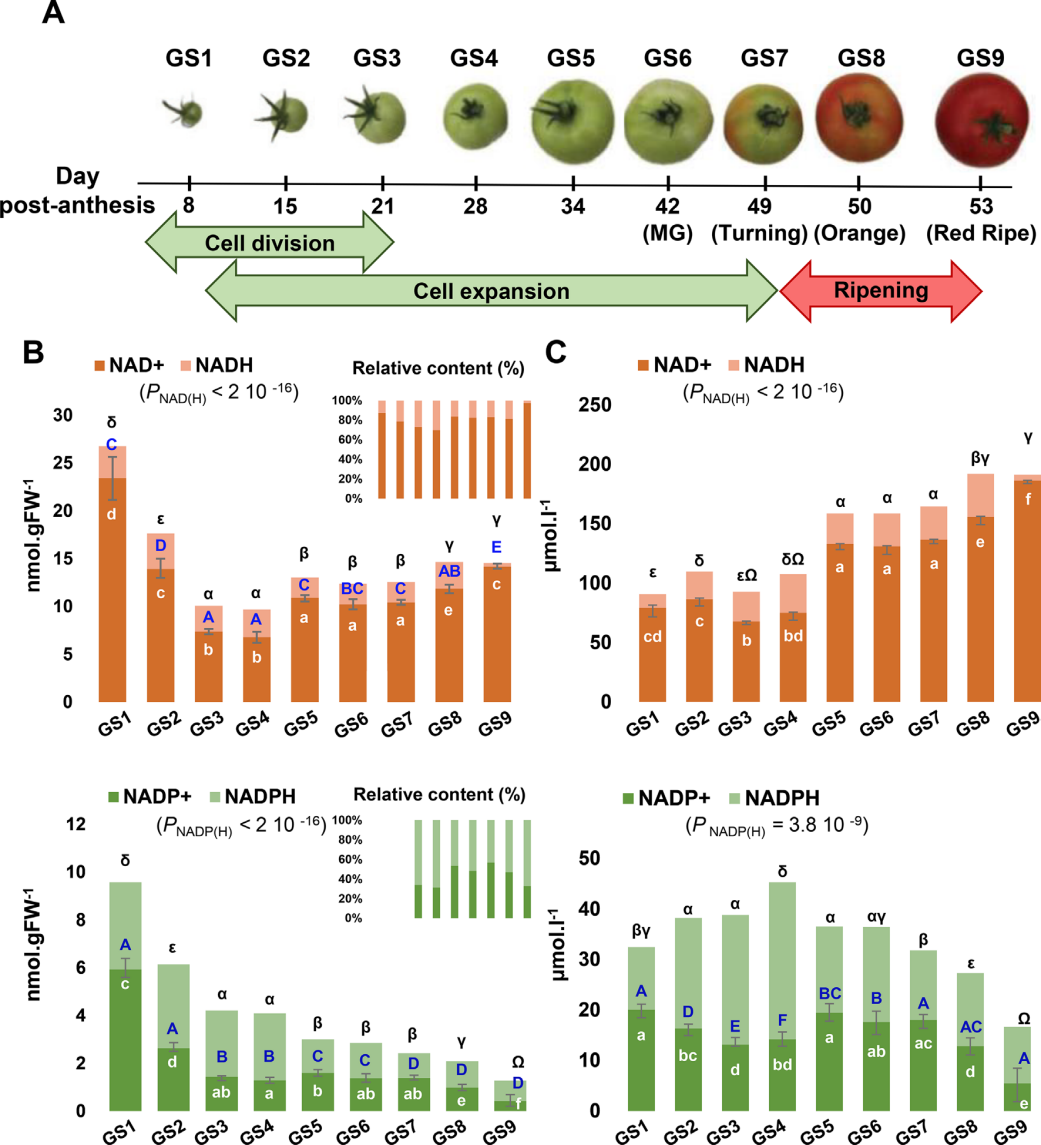
Evolution of NAD(P) contents during tomato fruit development. NAD and NADP pools were measured for nine sequential growth stages (GS) of tomato fruit development **(A)**. Shown are bar plots of replicated metabolite quantifications (*n* = 3) **(B)** and of replicated metabolite quantifications (*n* = 3) normalized to the cytosol and organelles volumes **(C)**. Top and bottom error bars indicate SEM (standard error of the mean) for the reduced and oxidized forms, respectively. Statistical significance for total NAD(P) content is indicated by ANOVA *P* value. Binary comparisons between conditions are indicated by letters (oxidized form, in white), capital letters (reduced form, in blue), and symbols (total content, in black), according to Student’s *t* test (*P* < 0.05). Left panel indicates the concentrations of NAD(P) in nmol.gFW^−1^ whereas the right panel indicates the NAD(P) concentrations in µmol.l^−1^.

Fruit development can be divided into three partially overlapping phases, namely, cell division, cell expansion, and ripening (**Figure 2A**), which all have their own metabolic specificity (Beauvoit et al., 2018). As a first attempt to clarify the importance of pyridine nucleotides for fruit growth, we measured total cellular NAD^+^, NADP^+^, NADH, and NADPH pools from nine growth stages of tomato fruit (var. Moneymaker) (**Figure 2B**). Oxidized pools were also confirmed by LCMS (**Figure S1**). Global changes in the pools of these pyridine nucleotides were statistically significant (ANOVA followed by binary Student’s *t* tests) and showed higher levels of both NAD(H) and NADP(H) in the very young fruit, with the highest pools observed at 8 days postanthesis (dpa) for growth stage 1 (GS1) and the lowest for mature green (GS6, 41 dpa) and for red ripe (GS9, 53 dpa) stages of tomato fruit, respectively. Reduced, oxidized, and total content of NAD(H) decreased until the end of cell division (GS4), then firstly increased during cell expansion (GS5–7) and secondly during ripening (GS8–9), while maintaining the NAD(H) pool mainly oxidized during fruit development. Since NAD(P) are mostly present in the chloroplasts, peroxisomes, mitochondria, and cytosol, but not in the vacuole (Donaldson, 1982; Gakière et al., 2018b), we could rule out the dilution effect due to the cell expansion (Beauvoit et al., 2014) and express NAD(P) contents as concentrations (**Figure 2C**). Differences in concentrations, except for NADH, were statistically significant during fruit growth (ANOVA followed by binary Student’s *t* tests) and clearly indicated two distinct waves of accumulation for NAD(H) (*i.e.*, GS5–7 and GS7–9). Simultaneously, NADP(H) only increased during the beginning of development (from GS1 to GS4) as a result of a higher NADPH concentration (**Figure 2C**). Hence, this suggests a fine-tuned NAD(P) homeostasis during tomato fruit development.

### Transcriptional Changes of NAD Biosynthesis and Metabolism Show Distinct Patterns During Tomato Fruit Growth

**Figure 3:**
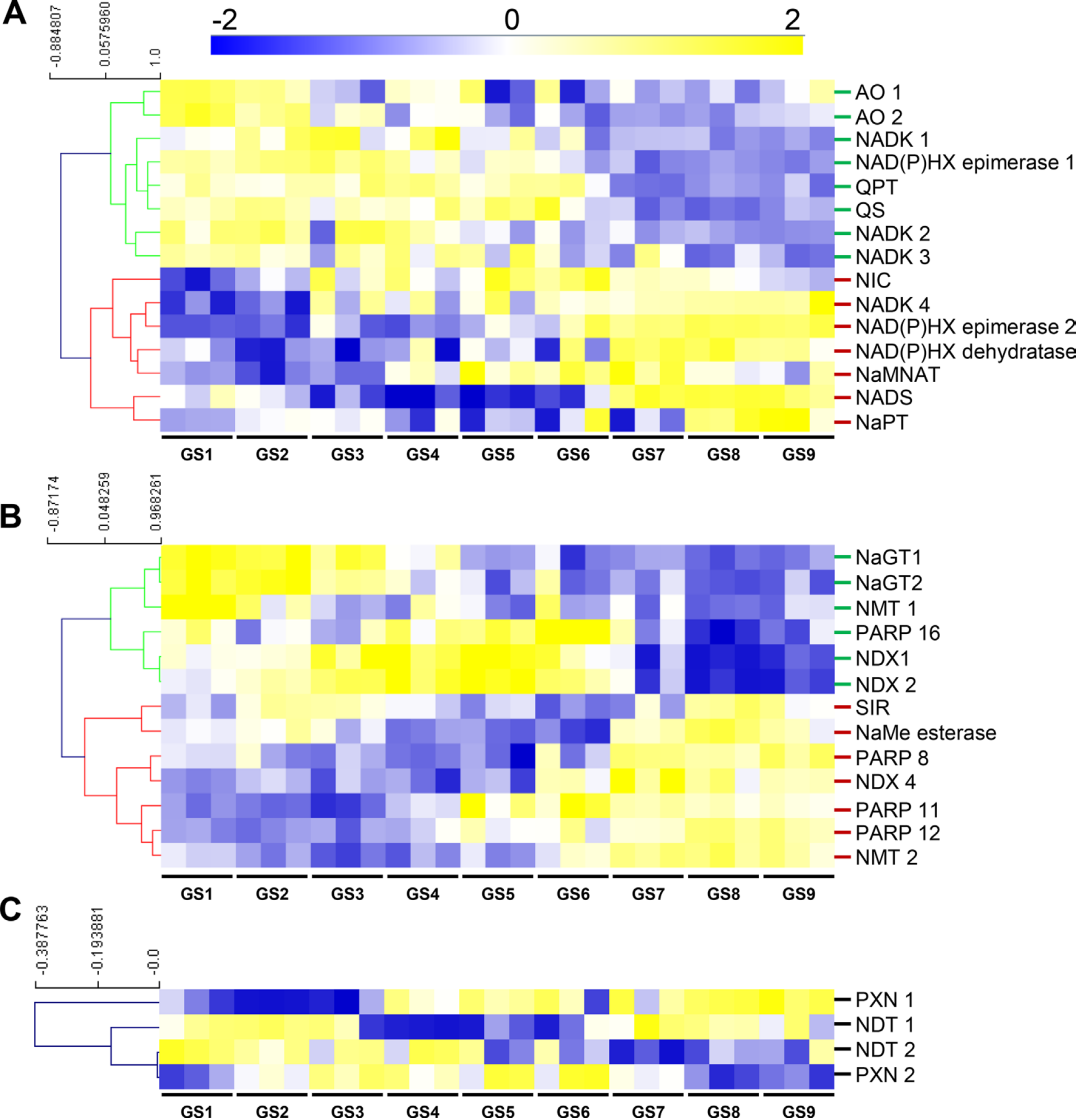
NAD^+^ synthesis **(A)**, consumption **(B)**, and transport **(C)** show transcriptional changes during tomato fruit development. Transcript data were normalized (see *Materials and Methods*) then filtered for statistically significant features (ANOVA with Bonferroni correction, *P* < 0.01) and subjected to clustering analysis using MeV (http://mev.tm4.org/). Shown are Pearson’s correlations after complete clustering of mRNA profiles. Names on the right refer to as enzymes of NAD^+^ synthesis **(A)**, consumption **(B)**, or putative transport **(C)**. NIC and NaPT were also found as significantly regulated during fruit growth for protein profiles (**Figure S2**). AO, aspartate oxidase; GS, growth stage; NADK, NAD kinase; NAD(P)HX, NAD(P)H hydrate; NaMNAT, NaMN adenylyltransferase; NAD(P)HX, NAD(P)H hydrate NADS, NAD synthetase; NaGT, nicotinate N-glucosyltransferase; NaPT, nicotinate phosphoribosyltransferase; NaMe, nicotinate methyl; NMT, nicotinate methyltransferase; NDT, nicotinamide adenine transporter; NDX, nudix; NIC, nicotinamidase; PARP, poly-ADP-ribose polymerase; PXN, peroxisomal NAD carrier; QPT, quinolinate phosphoribosyltransferase; QS, quinolinate synthase; SIR, sirtuin.

Next, in order to substantiate the changes in NAD(P), we examined transcript and protein profiles during tomato fruit development that were obtained from RNAseq and proteomics techniques (Belouah et al., 2019). Raw data (22,877 transcript and 2,375 protein features) were normalized as described previously (median-centered, cube root-transformed, and Pareto-scaled) (Belouah et al., 2019). We first focused on genes that were associated with NAD^+^ biosynthesis and NAD(P)H damage and repair (**Figure 1**), which included 15 genes (**Figure 3A**). Clustering analysis of mRNA profiles (Pearson’s correlation and complete clustering, **Figure 3A**) revealed two main statistically significant clusters (ANOVA with Bonferroni correction, *P* < 0.01 are listed in **Table S1**): one associated with the young fruit (stages 1–4) and another with the ripening fruit (stages 5–9). For the first cluster, early steps of *de novo* biosynthesis of NAD^+^ and NADP^+^ were transcriptionally up-regulated in the young fruit, including AO, QS and QPT, and NADK1 and NADK2 (**Figure 3A**). Interestingly, these enzymes are chloroplastic in *Arabidopsis* (Katoh et al., 2006) and assumed to be critical for NAD^+^ and NADP^+^ homeostasis, respectively (Pétriacq et al., 2012; Gakière et al., 2018a; Li et al., 2018a). For the second cluster, later stages of fruit growth coincided with the up-regulation of the expression of genes involved in final steps of NAD^+^ biosynthesis (NaMNAT and NADS), NAD^+^ recycling (NIC and NaPT), and NADP(H) production (NADK3 and NADK4) (**Figure 3A**). This suggests an accumulation of NAD^+^ precursors during the beginning of development (GS1–4) followed by an active synthesis and recycling of NAD(P)^+^ (GS5–9) concurrent with the increased NAD(H) content. Besides, NAD(P)XH epimerase and dehydratase genes are expressed all along fruit development suggesting a continuous NAD(P)H damage and repair (**Figure 3A**). Remarkably, protein profiles failed to include enzymes involved in NAD^+^ synthesis, except for NaPT and NIC that followed mRNA profiles with higher levels during ripening (**Figure S2**). This might suggest that most NAD metabolism enzymes were poorly represented quantitatively (*i.e.*, below threshold of protein detection). To test this hypothesis, we checked the absolute expression levels of genes involved in NAD^+^ synthesis as compared to other genes of central cellular functions (**Figure S3**). Raw data of mRNA profiles evidenced very low expression values for *AO* in tomato fruit, as compared to *NIC and NaPT*, and more remarkably, as compared to actin- and enolase-related genes (**Figure S3A**). Complementarily, data mining of expression profiles from published datasets (http://tea.solgenomics.net/; Fernandez-Pozo et al., 2017) confirmed that the NAD^+^ biosynthesis gene *AO* was expressed at low levels in fruit tissues as compared to other genes (**Figure S3B**), which can explain the absence of proteomic hits in our dataset.

Furthermore, we analyzed transcriptional changes of NAD^+^ catabolism involved in signaling and metabolism of nicotinate including 13 genes of PARPs, SIR, NDXs, NaGTs, NMTs, and NaMe esterase that demethylates NaMe into Na (**Figure 3B**) (Gakière et al., 2018b). Clustering analysis (Pearson’s correlation with complete clustering) unveiled three main significant clusters (ANOVA with Bonferroni correction, *P* < 0.01 are listed in **Table S1**). The young fruit correlated with an up-regulation of genes associated with NaGT and NMT functions, while older fruit (GS3–GS6) showed increased gene expression for PARP and NDX. The ripening fruit was associated with higher expression of genes associated with PARP, NMT, NaMe esterase, and NDX (**Figure 3B**).

Finally, we evaluated transcriptional changes of transporter genes that linked to putative pyridine nucleotide transport (**Figure 3C**). Transcript levels observed during early fruit development (GS1–4) correlated with the expression of NDT-like transporters suggesting an active transport of NAD(P) and its derivatives across the chloroplastic and mitochondrial membranes. Cell elongation (GS4–6) and ripening (GS7–9) phases were associated with higher expression of both PXN and NDT-like transporters, thus supporting the idea of an active NAD(P) transport during fruit development.

Altogether, RNAseq data demonstrate a profound reprogramming of NAD(P) metabolism during fruit growth, more specifically toward a stimulation of the synthesis of NAD^+^ precursors (*AO*, *QS*, and *QPT*) in chloroplast of young fruits, which was concurrent with the expression of NDT-like transporters. This was followed at later stages of fruit development by an active synthesis (*NaMNAT* and *NADS*) and recycling (*NIC* and *NaPT*) (**Figure 3**), concomitantly with increased NAD(H) pools and concentrations (**Figure 2**).

### Transcriptional Changes of Genes Associated to NAD(P)-Dependent Enzymes During Tomato Fruit Growth

**Figure 4:**
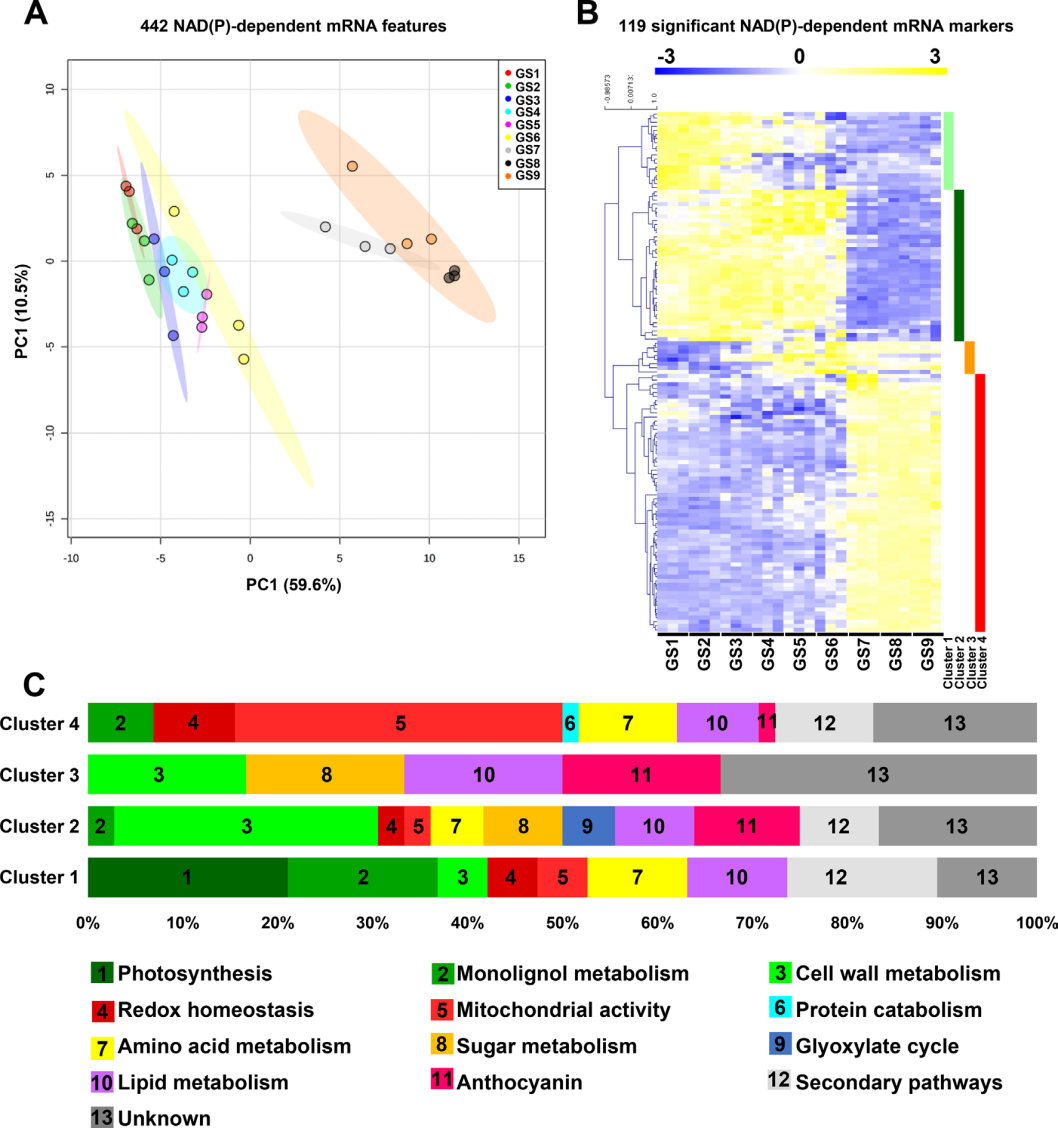
Expression of genes for NAD(P)-dependent enzymes reveals distinct clusters during tomato fruit growth. Normalized transcript data of 442 transcript features of NAD(P)-dependent enzyme (see Materials and Methods) were visualized **(A)** for global impact of the growth stage of tomato fruit by PCA (with maximal variation given into brackets). Same features were then filtered (ANOVA with Bonferroni correction, *P* < 0.01) and subjected to clustering analysis **(B)** using MeV (http://mev.tm4.org/). Shown are Pearson’s correlations after complete clustering of 119 significant mRNA profiles. Four clusters were identified and analyzed for functional classification based on their gene ontology annotations **(C)**. GS, growth stage.

To get a more global overview of the metabolic functions relating to pyridine nucleotides during tomato fruit growth, we analyzed the expression profiles of the genes that we could associate to NAD/P(H)-dependent functions. A selection of 442 NAD(P)-dependent features (see *Materials and Methods*) was subjected to PCA to display the global impact of growth stages on transcriptional changes for those features (**Figure 4A**). PCA explained 70% of the maximal variation in the mRNA profiles, where PC1 (59.6%) separated stages 1 to 6 from stages 7 to 9. This suggests a differential reprogramming at mRNA level of NAD(P)-dependent between cell division and expansion (GS1–6), and fruit ripening (GS7–9). Next, we filtered the same features for statistical significance (ANOVA with Bonferroni correction, *P* < 0.01 are listed in **Table S2**), which provided 119 mRNA markers that were retained for subsequent clustering analysis (Pearson’s correlation, complete clustering) (**Figure 4B**). Tomato fruit development was associated with four clusters distributed along the different growth stages, for which functional classification was performed based on gene ontology using Mercator4 v1.0 (https://plabipd.de/portal/mercator4; (Schwacke et al., 2019). This led to the identification of 13 different functional categories (**Figure 4C**) in which mitochondrial activity (18% of the total significant features) is the largest category represented for all growth stages. Cluster 1 corresponded to the beginning of tomato fruit development (GS1 to 5) and contained 19 genes mainly involved in energy supply for central metabolism, *i.e.*, photosynthesis (21%) and mitochondrial activity (5%). Cluster 1 further harbors several genes linked to central metabolism such as lipid-, sugar-, lignin-, and cell wall–related metabolisms (11, 10, 16, and 5%, respectively). Cluster 2 covered cell division and cell expansion stages of fruit growth and contained 36 genes mostly related to central metabolism [*i.e.*, cell wall, sugar, lipid, amino acid metabolism (28, 8, 8, 5%, respectively)] that are essential to cell proliferation by providing building blocks (**Figure 4C**). In addition, cluster 2 presented a substantial proportion (25%) of unknown functions or of genes involved in specialized metabolism (17 and 8%, respectively). Cluster 3 was represented only by six genes which were expressed from GS4 to GS6 (*i.e.*, the end of fruit expansion) and that were annotated to pigment synthesis, lipid, amino acid, and cell wall metabolisms (17% each) (**Figure 4C**). Cluster 4 contained the larger number of significant mRNA markers (58) that coded for genes mostly involved in mitochondrial activity (34%), but also the maintenance of redox homeostasis, lipid, amino acid, secondary pathways (9% each), and protein degradation (2%). Overall, gene expression for NAD(P)-dependent functions during fruit growth emphasizes the importance of pyridine nucleotides as cornerstones of central metabolism, which provides building blocks and energy for development. Importantly, NAD(P)-related mitochondrial functions seem crucial for fruit growth, more specifically at the onset of ripening.

### Protein Profiles for NAD(P)-Dependent Enzymes During Tomato Fruit Growth

**Figure 5:**
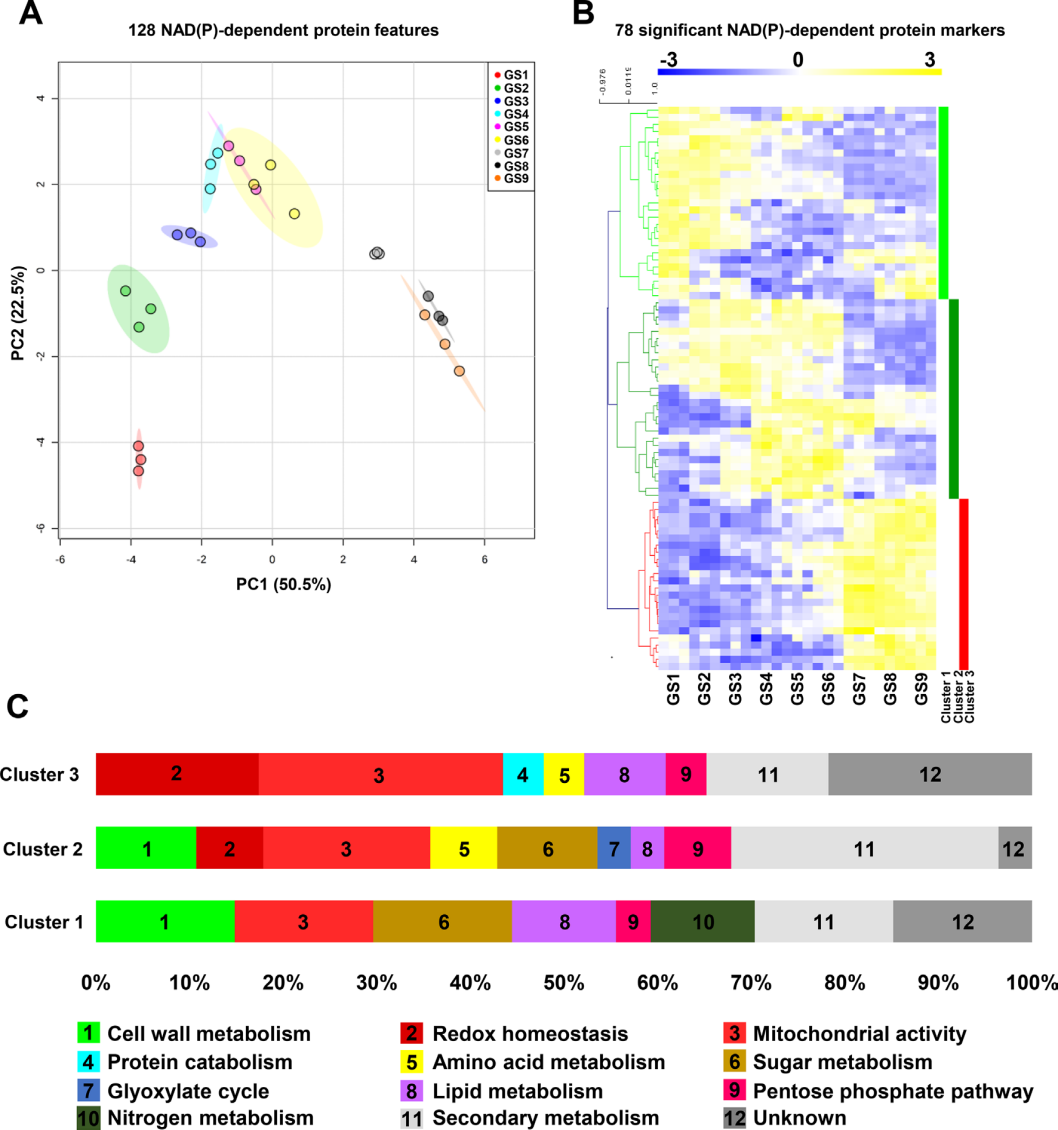
Protein profiles for NAD(P)-dependent enzymes unveil distinct clusters during tomato fruit growth. Normalized protein data 127 NAD(P)-dependent enzyme (see Materials and Methods) were visualized **(A)** for global impact of the growth stage of tomato fruit by principal component analysis (PCA with maximal variation given into brackets). Same features were then filtered (ANOVA with Bonferroni correction, *P* < 0.01) and subjected to clustering analysis **(B)** using MeV (http://mev.tm4.org/). Shown are Pearson’s correlations after complete clustering of 78 significant protein profiles. Four clusters were identified and analyzed for functional classification based on their gene ontology annotations **(C)**. GS, growth stage.

In addition to mRNA profiles, we further examined protein profiles for NAD(P)-dependent enzymes through global analysis by PCA of 128 protein features from normalized data, and by clustering of 78 significant protein markers (ANOVA with Bonferroni correction, *P* < 0.01 are listed in **Table S3**). In contrast to transcript signatures (**Figure 4A**), PCA better segregated the protein patterns according to GS1, GS2, and GS3 then merged GS4 to GS6, separated GS7, and gathered GS8 and GS9 (**Figure 5A**). This suggests that proteomics of NAD(P)-dependent enzymes is particularly sensitive to fruit growth. Complete clustering analysis by Pearson’s correlation resulted in three main clusters (**Figure 5B**) for which functional annotation of protein sequences was performed based on gene ontology using Mercator4 v1.0 leading to the identification of 12 different functional categories (**Figure 5C**) in which mitochondrial activity (19% of the total significant markers) was the largest category represented across all growth stages. To confirm the proteomic output, we measured enzyme activities of isocitrate dehydrogenase (NADP^+^-dependent) and malate dehydrogenase (NAD^+^-dependent) (**Figure S4**), which showed similar profile during fruit growth as compared to normalized protein concentration.

Firstly, cluster 1 is comprised of 27 NAD(P)-dependent proteins that accumulated mainly during the beginning of tomato fruit development (GS1–4) and during the ripening phases (GS7–9) for some of them (**Figure 5C**). This cluster could be divided into three segments; the first one contained enzymes involved in central metabolism (56%) such as cell wall-, sugar-, lipid-, and nitrogen-related metabolism and pentose phosphate pathway (15, 15, 11, 11, and 4%, respectively). The second portion is devoted to the energy production *via* the mitochondrial activity, constituting 15% of this cluster. The remaining part is represented by enzymes involved in specialized metabolism or unknown functions (15 and 15%, respectively) (**Figure 5C**). Secondly, cluster 2 included 28 proteins, which coincided with the fruit enlargement (GS1–6) within half of them that are stimulated during cell expansion (GS4–GS6). The most represented enzymes in this cluster are those involved in secondary pathways (29%) and mitochondrial functions (18%) (**Figure 5C**). Strikingly, central metabolism accounted for 42%, mainly because of cell wall and sugar metabolism (11% each) but also because of amino acid and lipid metabolism, glyoxylate cycle, and pentose phosphate pathway (7, 3, 3, and 7%, respectively) (**Figure 5C**). Finally, cluster 3 was constituted by 23 NAD(P)-dependent enzymes that were found during fruit ripening (from GS7 to GS9). Quite importantly, enzymes involved in mitochondrial activity were mainly represented (26%) in this last cluster, followed by proteins participating in redox homeostasis (18%) and secondary pathways (13%). Central metabolism (21%) remained noticeable due to the presence of enzymes involved in lipid and amino acid metabolisms, pentose phosphate pathway, and protein degradation (9, 4, 4, and 4%, respectively) (**Figure 5C**). Hence, while protein and mRNA profiles of NAD(P)-dependent markers only showed partial overlap during tomato fruit growth, central metabolism and more particularly mitochondrial functions were critically linked to pyridine nucleotides.

## Discussion

Pyridine nucleotides have received considerable attention for their metabolic, redox, and signaling functions in plant tissues (Hashida et al., 2009; Pétriacq et al., 2013; Gakière et al., 2018a; Gakière et al., 2018b). However, very little is known about the importance of these metabolic cofactors for fruit growth. In this study, we examined the contribution of pyridine nucleotides to fruit growth by analyzing NAD(P) metabolism at transcriptome and proteome scales as well as NAD(P)^+^ and NAD(P)H contents and concentrations during nine sequential stages of tomato fruit development.

Although previous changes in pyridine nucleotides have been observed between green and red tomato fruits of MicroTom and Moneymaker cultivars (Centeno et al., 2011; Osorio et al., 2013), our study presents for the first time the changes of NAD(P) contents and concentrations during nine sequential stages of tomato fruit development. As previously observed (Centeno et al., 2011), the highest contents (nmol.gFW^−1^) were measured during early developmental phases (**Figure 2**), concomitantly with the expression of genes involved in early reactions of NAD^+^ *de novo* synthesis (AO, QS, and QPT) (**Figure 3**), also assumed to control critically NAD(P) levels and its derivatives (Pétriacq et al., 2012; Pétriacq et al., 2016). Firstly, NAD(H) contents and concentrations showed two distinct accumulations mainly caused by increased NAD^+^ at the beginning of cell elongation and ripening (GS5 and GS8) (**Figure 2**). Concurrently, transcript analysis unveiled a stimulation of further steps of NAD^+^ *de novo* synthesis and recycling pathway (**Figure 3A**). Secondly, NADP(H) contents dropped during cell division (GS1–4) whereas concentrations were augmented as a result of increased NADPH concentrations. Furthermore, cell expansion and ripening phases displayed a decrease in both NADP(H) contents and concentrations. Since NAD(P) are mostly present in organelles other than the vacuole (Donaldson, 1982; Gakière et al., 2018b), concentrations seem more relevant to understand the involvement of NADP(H) in fruit development (**Figure 2C**). However, pyridine nucleotide concentrations that are reported here and in previous studies represent pooled contents of subcellular metabolites rather than the *in vivo* original compartmentalized concentrations, which are known to differ between organelles (Gakière et al., 2018b). Such differences in subcellular concentrations might affect the enzyme activities that depend on pyridine nucleotides as cofactor. Meta-analysis of *K_m_* values for plant dehydrogenases (Bar-Even et al., 2011) showed that total cellular concentrations of NAD(P) measured in our study were higher than the median *K_m_* values of plant NAD(P)-dependent enzymes (**Figure S5**). This indicates that concentrations of pyridine nucleotides in the developing fruit are sufficiently high that they would not limit NAD(P)-dependent enzyme activities, as previously suggested (Bennett et al., 2009). In growing tissues, such as *Arabidopsis* pollen tubes (Hashida et al., 2012), or here in young tomato fruit that is characterized by a *turbo* metabolism due to high enzyme activities (Biais et al., 2014), plant development requires higher NAD^+^ and reducing power (NADPH) (Gakière et al., 2018b). High concentrations of NADP(H) in young fruits (**Figure 2C**) might be associated with photosynthesis that remains active in green fruits, as detected in our transcript analysis (**Figure 4C**) and previously reported (Lytovchenko et al., 2011). Furthermore, mRNA and protein analyses indicated a primary accumulation of NAD^+^ precursors (GS1–4) before the activation of *de novo* and recycling (GS4–9) pathways during tomato fruit growth (**Figure 3**), which correlated with both contents and concentrations of NAD(H). Interestingly, the stimulation of NaMNAT and NADS at transcript levels (**Figure 3A**) occurred just before the increase in NAD(H) concentrations (**Figure 2C**). On the other hand, catabolism of NAD(P) *via* signaling functions appeared continuous during fruit development but resulted from different catabolic pathways (**Figure 3B**) as well as transport of NAD(P) and their precursors (**Figure 3C**). This indicates that NAD(P) contents are sustained as key metabolic regulators for fruit growth by different synthesis and degradation pathways. Precursors are synthesized by early enzymes of *de novo* synthesis of NAD^+^ (AO, QS, QPT); then, latter enzymes of synthesis and salvage routes (NaMNAT, NADS, NIC, NaPT, NaGT, and NAD(P)H-hydrate epimerase and hydratase) (**Figure 1**) allow for NAD(P) homeostasis. This agrees with previous works which have demonstrated the importance of AO, QS, and QPT in modulating NAD^+^ levels that influence plant development (Katoh et al., 2006; Schippers et al., 2008; Pétriacq et al., 2012; Gakière et al., 2018a). Likewise, *Arabidopsis* NICs were also reported to influence NAD^+^ contents (Hunt et al., 2007; Wang and Pichersky, 2007). Metabolism of nicotinate has also received recent attention in various plant species (Matsui et al., 2007; Li et al., 2015; Wu et al., 2018). In this context, we observed increased expression of NMT and NaMe esterase genes in the developing fruit (**Figure 3**), suggesting that Na metabolism is important to sustain NAD(P) homeostasis. Nicotinate conjugates are particularly difficult to measure *via* MS-based metabolomics due to unreliable ionizations of such compounds in the mass spectrometer, as it is the case for NAD^+^ and nicotinamide (Guérard et al., 2011). Further research combining MS and NMR techniques is necessary to confirm the molecular and regulatory details of nicotinate metabolism in fruit.

In all biological systems, a plethora of cellular processes need NAD(P) as coenzymes, including both biosynthetic pathways and the catabolism of biomolecules which support energy production (Gakière et al., 2018a). Mitochondria are the powerhouse of the cell that ensure energy supply by regenerating NADH and providing ATP and are considered to contain the highest proportion of cellular NAD(H). Several studies have shown that modifying mitochondrial functions result in changes of NAD(P) contents that not only greatly influence plant growth (Dutilleul, 2003; Dutilleul et al., 2003; Dutilleul, 2005; Pellny et al., 2008; Meyer et al., 2009; Centeno et al., 2011; Osorio et al., 2013; Pétriacq et al., 2017) but also stress responses (Pétriacq et al., 2016; Gakière et al., 2018b), thus placing pyridine nucleotides as critical regulators of plant performance. In line with this, we demonstrated that NAD(P)-dependent enzymes appeared mainly related to central metabolic pathways, including one-fifth of mitochondrial functions (**Figures 4C** and **5C**). This agrees with a critical role of mitochondrial NAD(P) metabolism for tomato fruit growth, as elegantly demonstrated in mutant tomato fruit that are affected in TCA cycle (Araújo et al., 2012). Additionally, we showed that mRNAs and proteins that related to NAD(P)-dependent enzymes exhibited distinguishable profiles during fruit development. We further identified a global separation between ripening and previous developmental stages (*i.e.*, cell division and expansion) (**Figures 4A** and **5A**), based on the profiles of both NAD(P)-dependent mRNAs and proteins. This suggests a growth stage-dependent reprogramming of NAD(P) metabolism. Concurrently, we observed a growth stage-dependent stimulation of the different central metabolism branches (amino acid-, lipid-, sugar-, and cell wall–related metabolism) at both transcript and proteomic levels (**Figures 4C** and **5C**), thus emphasizing the link between NAD(P) and carbon and nitrogen metabolisms. This raises the question about NAD(P) signaling during fruit development (Hashida et al., 2018), as further exemplified by a notable proportion of NAD(P)-dependent enzymes that related to redox homeostasis at mRNA and protein levels, especially those involved in ascorbate–glutathione cycle, and other specialized pathways (e.g., anthocyanins biosynthesis). Here, we showed that cellular division phases displayed an increase in reducing power equivalent (NADPH) while cellular expansion and ripening phases harbored a higher oxidized state. Hence, pyridine nucleotides appear pivotal for fruit development in supplying energy and precursors for central metabolism while assuring redox homeostasis and secondary metabolites accumulation. For a better comprehension of NAD(P) involvement in cellular processes, future works should also include the study of protein turnover, especially protein synthesis rate that has been shown to control protein levels in developing tomato fruit (Belouah et al., 2019).

Climacteric species (e.g., tomato, banana, kiwi.) are characterized by a metabolic shift from normal development state, known as climacteric crisis, that is associated with the conversion of starch into soluble sugars and CO_2_ (Moing et al., 2001; Colombié et al., 2017), and which is concomitant with higher ATP levels and respiratory fluxes. Interestingly, from a transcript perspective, 50% of the significant NAD(P)-dependent genes that were identified as up-regulated during ripening belonged to enzymes involved in the key respiratory burst during ripening of climacteric fruits (**Figure 4**) in accordance with previous reports (Biais et al., 2014; Colombié et al., 2015; Colombié et al., 2017). Moreover, mitochondrial functions represented a notable proportion of both protein and transcript markers, and more specifically, a greater proportion (>75%) within the NAD(P)-dependent ripening-related markers, thus supporting the idea that climacteric respiration is *(i)* regulated by mitochondrial activity and *(ii)* essential for fruit ripening (**Figures 4B, C**). In tomato fruit, the energy peak results from an excessive carbon supply coming from starch and cell wall degradation, and a decrease in carbon demand as a result of arrested cell expansion (Colombié et al., 2017). In agreement, several sugar- and cell wall–related NAD(P)-dependent markers are identified to be up-regulated shortly before and during ripening (**Figures 4** and **5**). Besides, NAD(P)-dependent markers involved in redox homeostasis were also up-regulated mainly during ripening (**Figures 4** and **5**). Despite the energy peak and the control of redox balance, ripening is also associated with a wide range of metabolic processes resulting in organoleptic changes reflected by an increased sweetness, and nutritional value. This further agrees with a concurrent accumulation of soluble sugars and others amino acids coming from starch and protein degradation, respectively (Beauvoit et al., 2018). Here, functional clustering identified an increase of NAD(P)-dependent transcript and protein levels involved in protein catabolism only during ripening phases as well as amino acid-related metabolism markers (**Figures 4** and **5**). Finally, latter stages of NAD^+^ synthesis and recycling were stimulated during ripening at both transcript and protein levels (**Figure 3**) that resulted in an augmented NAD(H) concentrations (**Figure 2**), probably to sustain the high metabolic activity during fruit-ripening process. Collectively, these results indicate that NAD(P) is a core component of tomato fruit ripening, which not only participates in the climacteric respiration *via* a stimulation of mitochondrial activity but also sustains energy supply for numerous biosynthetic pathways that relate pyridine nucleotide metabolism to fruit development and quality.

## Conclusion

This study is a first step toward a better comprehension of the implication of NAD(P) in fruit development. Our results demonstrate a crucial role of NAD(P) during the whole process of fruit growth with distinct functions between the cellular division, expansion, and ripening stages. Further experiments are required to decipher which biochemical and molecular mechanisms are triggered by pyridine nucleotides and participate in the control of fruit development. Due to the high reactivity of such redox metabolites, metabolic modeling tools might provide a great alternative to predict fluxes of NAD(P) during fruit development and a better understanding of these mechanisms that will help to improve fruit performance, thus allowing better strategies for crop productions.

## Data Availability Statement

The datasets generated for this study can be found in https://www.ncbi.nlm.nih.gov/geo/query/acc.cgi?acc=GSE128739, GSE128739.

## Author Contributions

PP designed the work with inputs from GD, BB, SC, and YG. GD, IB, BB, SB, CC, SA, and MG performed the experiments. GD, BB, PP, SP and PB analysed the data with substantial data interpretation from all the authors. GD and PP wrote the manuscript with inputs from all the authors.

## Funding

The authors are grateful for financial support from INRA BAP, University of Bordeaux and to the FRIMOUSS (ANR-15-CE20-0009-01), MetaboHUB (ANR-11-INBS-0010) and PHENOME (ANR-11-INBS-0012) projects. The doctoral school *Sciences de la Vie et Santé* at Université de Bordeaux is also acknowledged for granting PP with PhD funding for GD (bourse fléchée ministérielle 2018-2021).

## Conflict of Interest

The authors declare that the research was conducted in the absence of any commercial or financial relationships that could be construed as a potential conflict of interest.

## References

[B1] Alferez F. M. Gerberich K. M. Li J.-L. Zhang Y. Graham J. H. Mou Z. (2018). Exogenous nicotinamide adenine dinucleotide induces resistance to citrus canker in citrus. Front. Plant Sci. 9, 1472. 10.3389/fpls.2018.01472 30356715 PMC6189366

[B2] Araújo W. L. Nunes-Nesi A. Nikoloski Z. Sweetlove L. J. Fernie A. R. (2012). Metabolic control and regulation of the tricarboxylic acid cycle in photosynthetic and heterotrophic plant tissues: TCA control and regulation in plant tissues. Plant Cell Environ. 35, 1–21. 10.1111/j.1365-3040.2011.02332.x 21477125

[B3] Arrivault S. Guenther M. Ivakov A. Feil R. Vosloh D. van Dongen J. T. (2009). Use of reverse-phase liquid chromatography, linked to tandem mass spectrometry, to profile the Calvin cycle and other metabolic intermediates in *Arabidopsis* rosettes at different carbon dioxide concentrations. Plant J. 59, 826–839. 10.1111/j.1365-313X.2009.03902.x 19453453

[B4] Bar-Even A. Noor E. Savir Y. Liebermeister W. Davidi D. Tawfik D. S. (2011). The moderately efficient enzyme: evolutionary and physicochemical trends shaping enzyme parameters. Biochemistry 50, 4402–4410. 10.1021/bi2002289 21506553

[B5] Barrett T. Wilhite S. E. Ledoux P. Evangelista C. Kim I. F. Tomashevsky M. (2013). NCBI GEO: archive for functional genomics data sets—update. Nucleic Acids Res. 41, 991–995. 10.1093/nar/gks1193 PMC3531084 23193258

[B6] Beauvoit B. Belouah I. Bertin N. Cakpo C. B. Colombié S. Dai Z. (2018). Putting primary metabolism into perspective to obtain better fruits. Ann. Bot. 122, 1–21. 10.1093/aob/mcy057 29718072 PMC6025238

[B7] Beauvoit B. P. Colombié S. Monier A. Andrieu M.-H. Biais B. Bénard C. (2014). Model-assisted analysis of sugar metabolism throughout tomato fruit development reveals enzyme and carrier properties in relation to vacuole expansion. Plant Cell 26, 3224–3242. 10.1105/tpc.114.127761 25139005 PMC4371827

[B8] Belouah I. Nazaret C. Pétriacq P. Prigent S. Bénard C. Mengin V. (2019). Modeling protein destiny in developing fruit. Plant Physiol. 190, 1709–1724. 10.1104/pp.19.00086 PMC6752906 31015299

[B9] Bennett B. D. Kimball E. H. Gao M. Osterhout R. Van Dien S. J. Rabinowitz J. D. (2009). Absolute metabolite concentrations and implied enzyme active site occupancy in *Escherichia coli* . Nature Chem. Biol. 5, 593–599. 10.1038/nchembio.186 19561621 PMC2754216

[B10] Biais B. Bénard C. Beauvoit B. Colombié S. Prodhomme D. Ménard G. (2014). Remarkable reproducibility of enzyme activity profiles in tomato fruits grown under contrasting environments provides a roadmap for studies of fruit metabolism. Plant Physiol. 164, 1204–1221. 10.1104/pp.113.231241 24474652 PMC3938614

[B11] Bowsher C. G. Lacey A. E. Hanke G. T. Clarkson D. T. Saker L. R. Stulen I. (2007). The effect of Glc6P uptake and its subsequent oxidation within pea root plastids on nitrite reduction and glutamate synthesis. J. Exp. Bot. 58, 1109–1118. 10.1093/jxb/erl269 17220512

[B12] Briggs A. G. Bent A. F. (2011). Poly(ADP-ribosyl)ation in plants. Trends Plant Sci. 16, 372–380. 10.1016/j.tplants.2011.03.008 21482174

[B13] Centeno D. C. Osorio S. Nunes-Nesi A. Bertolo A. L. F. Carneiro R. T. Araújo W. L. (2011). Malate plays a crucial role in starch metabolism, ripening, and soluble solid content of tomato fruit and affects postharvest softening. Plant Cell 23, 162–184. 10.1105/tpc.109.072231 21239646 PMC3051241

[B14] Colombié S. Beauvoit B. Nazaret C. Bénard C. Vercambre G. Le Gall S. (2017). Respiration climacteric in tomato fruits elucidated by constraint-based modelling. New Phytol. 213, 1726–1739. 10.1111/nph.14301 27861943 PMC6079640

[B15] Colombié S. Nazaret C. Bénard C. Biais B. Mengin V. Solé M. (2015). Modelling central metabolic fluxes by constraint-based optimization reveals metabolic reprogramming of developing *Solanum lycopersicum* (tomato) fruit. Plant J. 81, 24–39. 10.1111/tpj.12685 25279440 PMC4309433

[B16] Donaldson R. P. (1982). Nicotinamide cofactors (NAD and NADP) in glyoxysomes, mitochondria, and plastids isolated from castor bean endosperm. Arch. Biochem. Biophys. 215, 274–279. 10.1016/0003-9861(82)90305-8 7092229

[B17] Dutilleul C. (2003). Leaf mitochondria modulate whole cell redox homeostasis, set antioxidant capacity, and determine stress resistance through altered signaling and diurnal regulation. Plant Cell 15, 1212–1226. 10.1105/tpc.009464 12724545 PMC153727

[B18] Dutilleul C. (2005). Mitochondria-driven changes in leaf NAD status exert a crucial influence on the control of nitrate assimilation and the integration of carbon and nitrogen metabolism. Plant Physiol. 139, 64–78. 10.1104/pp.105.066399 16126851 PMC1203358

[B19] Dutilleul C. Driscoll S. Cornic G. Paepe R. D. Foyer C. H. Noctor G. (2003). Functional mitochondrial complex I is required by tobacco leaves for optimal photosynthetic performance in photorespiratory conditions and during transients. Plant Physiol. 131, 264–275. 10.1104/pp.011155 12529534 PMC166806

[B20] Faurobert M. Pelpoir E. Chaïb J. (2007). Phenol extraction of proteins for proteomic studies of recalcitrant plant tissues. Methods Mol. Biol. 355, 9–14. 10.1385/1-59745-227-0:9 17093297

[B21] Fernandez-Pozo N. Zheng Y. Snyder S. I. Nicolas P. Shinozaki Y. Fei Z. (2017). The tomato expression atlas. Bioinformatics 33, 2397–2398. 10.1093/bioinformatics/btx190 28379331 PMC5860121

[B22] Gakière B. Fernie A. R. Pétriacq P. (2018a). More to NAD+ than meets the eye: a regulator of metabolic pools and gene expression in *Arabidopsis* . Free Radic. Biol. Med. 122, 86–95. 10.1016/j.freeradbiomed.2018.01.003 29309893

[B23] Gakière B. Hao J. de Bont L. Pétriacq P. Nunes-Nesi A. Fernie A. R. (2018b). NAD + biosynthesis and signaling in plants. Crit. Rev. Plant Sci. 37, 1–49. 10.1080/07352689.2018.1505591

[B24] Geigenberger P. Fernie A. R. (2014). Metabolic control of redox and redox control of metabolism in plants. Antioxid. Redox Signal. 21, 1389–1421. 10.1089/ars.2014.6018 24960279 PMC4158967

[B25] Gibon Y. Blaesing O. E. Hannemann J. Carillo P. Höhne M. Hendriks J. H. M. (2004). A robot-based platform to measure multiple enzyme activities in *Arabidopsis* using a set of cycling assays: comparison of changes of enzyme activities and transcript levels during diurnal cycles and in prolonged darkness. Plant Cell 16, 3304–3325. 10.1105/tpc.104.025973 15548738 PMC535875

[B26] Gibon Y. Pyl E.-T. Sulpice R. Lunn J. E. Höhne M. Günther M. (2009). Adjustment of growth, starch turnover, protein content and central metabolism to a decrease of the carbon supply when *Arabidopsis* is grown in very short photoperiods. Plant Cell Environ. 32, 859–874. 10.1111/j.1365-3040.2009.01965.x 19236606

[B27] Gibon Y. Usadel B. Blaesing O. E. Kamlage B. Hoehne M. Trethewey R. (2006). Integration of metabolite with transcript and enzyme activity profiling during diurnal cycles in *Arabidopsis* rosettes. Genome Biol. 7, R76. 10.1186/gb-2006-7-8-R76 16916443 PMC1779593

[B28] Guérard F. Pétriacq P. Gakière B. Tcherkez G. (2011). Liquid chromatography/time-of-flight mass spectrometry for the analysis of plant samples: a method for simultaneous screening of common cofactors or nucleotides and application to an engineered plant line. Plant Physiol. Biochem. 49, 1117–1125. 10.1016/j.plaphy.2011.06.003 21723140

[B29] Hashida S. N. Takahashi H. Uchimiya H. (2009). The role of NAD biosynthesis in plant development and stress responses. Ann. Bot. 103, 819–824. 10.1093/aob/mcp019 19201765 PMC2707885

[B30] Hashida S. Takahashi H. Kawai-Yamada M. Uchimiya H. (2007). *Arabidopsis thaliana* nicotinate/nicotinamide mononucleotide adenyltransferase (AtNMNAT) is required for pollen tube growth: AtNMNATis required for pollen tube growth. Plant J. 49, 694–703. 10.1111/j.1365-313X.2006.02989.x 17270012

[B31] Hashida S.-N. Miyagi A. Nishiyama M. Yoshida K. Hisabori T. Kawai-Yamada M. (2018). Ferredoxin/thioredoxin system plays an important role in the chloroplastic NADP status of *Arabidopsis* . Plant J. 95, 947–960. 10.1111/tpj.14000 29920827

[B32] Hashida S.-N. Takahashi H. Takahara K. Kawai-Yamada M. Kitazaki K. Shoji K. (2012). NAD+ Accumulation during Pollen maturation in *arabidopsis* regulating onset of germination. Mol. Plant 6, 216–225. 10.1093/mp/sss071 22907882

[B33] Havé M. Balliau T. Cottyn-Boitte B. Dérond E. Cueff G. Soulay F. (2018). Increases in activity of proteasome and papain-like cysteine protease in *Arabidopsis* autophagy mutants: back-up compensatory effect or cell-death promoting effect? J. Exp. Bot. 69, 1369–1385. 10.1093/jxb/erx482 29281085 PMC6037082

[B34] Hunt L. Gray J. E. (2009). The relationship between pyridine nucleotides and seed dormancy. New Phytol. 181, 62–70. 10.1111/j.1469-8137.2008.02641.x 18826484

[B35] Hunt L. Holdsworth M. J. Gray J. E. (2007). Nicotinamidase activity is important for germination. Plant J. 51, 341–351. 10.1111/j.1365-313X.2007.03151.x 17587307

[B36] Hunt L. Lerner F. Ziegler M. (2004). NAD—new roles in signalling and gene regulation in plants. New Phytol. 163, 31–44. 10.1111/j.1469-8137.2004.01087.x 33873776

[B37] Katoh A. Uenohara K. Akita M. Hashimoto T. (2006). Early steps in the biosynthesis of NAD in *Arabidopsis* start with aspartate and occur in the plastid. Plant Physiol. 141, 851–857. 10.1104/pp.106.081091 16698895 PMC1489895

[B38] Köster S. Upmeier B. Komossa D. Barz W. (1989). Nicotinic acid conjugation in plants and plant cell cultures of potato (*Solanum tuberosum*). Z. Naturforsch. C. 44, 623–628. 10.1515/znc-1989-7-813

[B39] Kraszewska E. (2008). The plant Nudix hydrolase family. Acta Biochim. Pol. 55, 663–671. 19081844 19081844

[B40] Kupis W. Pałyga J. Tomal E. Niewiadomska E. (2016). The role of sirtuins in cellular homeostasis. J. Physiol. Biochem. 72, 371–380. 10.1007/s13105-016-0492-6 27154583 PMC4992043

[B41] Li B.-B. Wang X. Tai L. Ma T.-T. Shalmani A. Liu W.-T. (2018a). NAD kinases: metabolic targets controlling redox co-enzymes and reducing power partitioning in plant stress and development. Front. Plant Sci. 9, 379. 10.3389/fpls.2018.00379 29662499 PMC5890153

[B42] Li W. Zhang F. Chang Y. Zhao T. Schranz M. E. Wang G. (2015). Nicotinate O-glucosylation is an evolutionarily metabolic trait important for seed germination under stress conditions in *Arabidopsis thaliana* . Plant Cell 27, 1907–1924. 10.1105/tpc.15.00223 26116607 PMC4531354

[B43] Li W. Zhang F. Wu R. Jia L. Li G. Guo Y. (2017). A novel N-methyltransferase in *arabidopsis* appears to feed a conserved pathway for nicotinate detoxification among land plants and is associated with lignin biosynthesis. Plant Physiol. 174, 1492–1504. 10.1104/pp.17.00259 28533213 PMC5490898

[B44] Li W.-Y. Wang X. Li R. Li W.-Q. Chen K.-M. (2014). Genome-wide analysis of the NADK gene family in plants. PLoS One 9 (6), e101051. 10.1371/journal.pone.0101051 24968225 PMC4072752

[B45] Li Y. Wang H. Zhang Y. Martin C. (2018b). Can the world’s favorite fruit, tomato, provide an effective biosynthetic chassis for high-value metabolites? Plant Cell Rep. 37, 1443–1450. 10.1007/s00299-018-2283-8 29594330 PMC6153642

[B46] Lytovchenko A. Eickmeier I. Pons C. Osorio S. Szecowka M. Lehmberg K. (2011). Tomato fruit photosynthesis is seemingly unimportant in primary metabolism and ripening but plays a considerable role in seed development. Plant Physiol. 157, 1650–1663. 10.1104/pp.111.186874 21972266 PMC3327185

[B47] Macho A. P. Boutrot F. Rathjen J. P. Zipfel C. (2012). Aspartate oxidase plays an important role in *Arabidopsis* stomatal immunity. Plant Physiol. 159, 1845–1856. 10.1104/pp.112.199810 22730426 PMC3425217

[B48] Magni G. Amici A. Emanuelli M. Orsomando G. Raffaelli N. Ruggieri S. (2004). Enzymology of NAD+ homeostasis in man. Cell. Mol. Life Sci. 61, 19–34. 10.1007/s00018-003-3161-1 14704851 PMC11138864

[B49] Mahalingam R. Jambunathan N. Penaganti A. (2007). Pyridine nucleotide homeostasis in plant development and stress. Int. J. Plant Dev. Biol. 1, 194–201.

[B50] Matsui A. Yin Y. Yamanaka K. Iwasaki M. Ashihara H. (2007). Metabolic fate of nicotinamide in higher plants. Physiol. Plant 131, 191–200. 10.1111/j.1399-3054.2007.00959.x 18251891

[B51] Meyer E. H. Tomaz T. Carroll A. J. Estavillo G. Delannoy E. Tanz S. K. (2009). Remodeled respiration in ndufs4 with low phosphorylation efficiency suppresses *Arabidopsis* germination and growth and alters control of metabolism at night. Plant Physiol. 151, 603–619. 10.1104/pp.109.141770 19675153 PMC2754622

[B52] Moing A. Renaud C. Gaudillère M. Raymond P. Roudeillac P. Denoyes-Rothan B. (2001). Biochemical changes during fruit development of four strawberry cultivars. J. Am. Soc. Hortic. Sci. 126, 394–403. 10.21273/JASHS.126.4.394

[B53] Mou Z. (2017). Extracellular pyridine nucleotides as immune elicitors in *Arabidopsis* . Plant Signal Behav. 12. 10.1080/15592324.2017.1388977 PMC5703255 29035673

[B54] Neuhaus H. E. Emes M. J. (2000). Nonphotosynthetic metabolism in plastids. Annu. Rev. Plant Physiol. Plant Mol. Biol. 51, 111–140. 10.1146/annurev.arplant.51.1.111 15012188

[B55] Niehaus T. D. Richardson L. G. L. Gidda S. K. Badawi-Sidhu M. Meissen J. K. Mullen R. T. (2014). Plants utilize a highly conserved system for repair of NADH and NADPH Hydrates. Plant Physiol. 165, 52–61. 10.1104/pp.114.236539 24599492 PMC4012604

[B56] Noctor G. Queval G. Gakière B. (2006). NAD(P) synthesis and pyridine nucleotide cycling in plants and their potential importance in stress conditions. J. Exp. Bot. 57, 1603–1620. 10.1093/jxb/erj202 16714307

[B57] Osorio S. Vallarino J. G. Szecowka M. Ufaz S. Tzin V. Angelovici R. (2013). Alteration of the interconversion of pyruvate and malate in the plastid or cytosol of ripening tomato fruit invokes diverse consequences on sugar but similar effects on cellular organic acid, metabolism, and transitory starch accumulation. Plant Physiol. 161, 628–643. 10.1104/pp.112.211094 23250627 PMC3561009

[B58] Pellny T. K. Aken O. V. Dutilleul C. Wolff T. Groten K. Bor M. (2008). Mitochondrial respiratory pathways modulate nitrate sensing and nitrogen-dependent regulation of plant architecture in Nicotiana sylvestris. Plant J. 54, 976–992. 10.1111/j.1365-313X.2008.03472.x 18318685 PMC2440565

[B59] Perez-Riverol Y. Csordas A. Bai J. Bernal-Llinares M. Hewapathirana S. Kundu D. J. (2019). The PRIDE database and related tools and resources in 2019: improving support for quantification data. Nucleic Acids Res. 47, D442–D450. 10.1093/nar/gky1106 30395289 PMC6323896

[B60] Pétriacq P. de Bont L. Genestout L. Hao J. Laureau C. Florez-Sarasa I. (2017). Photoperiod affects the phenotype of mitochondrial complex I mutants. Plant Physiol. 173, 434–455. 10.1104/pp.16.01484 27852950 PMC5210746

[B61] Pétriacq P. de Bont L. Hager J. Didierlaurent L. Mauve C. Guérard F. (2012). Inducible NAD overproduction in *Arabidopsis* alters metabolic pools and gene expression correlated with increased salicylate content and resistance to Pst-AvrRpm1. Plant J. 70, 650–665. 10.1111/j.1365-313X.2012.04920.x 22268572

[B62] Pétriacq P. de Bont L. Tcherkez G. Gakière B. (2013). NAD: not just a pawn on the board of plant-pathogen interactions. Plant Signal Behav. 8, 1–11. 10.4161/psb.22477 PMC3745554 23104110

[B63] Pétriacq P. Ton J. Patrit O. Tcherkez G. Gakière B. (2016). NAD acts as an integral regulator of multiple defense layers. Plant Physiol. 172, 1465–1479. 10.1104/pp.16.00780 27621425 PMC5100754

[B64] Queval G. Noctor G. (2007). A plate reader method for the measurement of NAD, NADP, glutathione, and ascorbate in tissue extracts: application to redox profiling during *Arabidopsis* rosette development. Anal. Biochem. 363, 58–69. 17288982 10.1016/j.ab.2007.01.005

[B65] Schippers J. H. M. Nunes-Nesi A. Apetrei R. Hille J. Fernie A. R. Dijkwel P. P. (2008). The *Arabidopsis* onset of leaf death5 mutation of quinolinate synthase affects nicotinamide adenine dinucleotide biosynthesis and causes early ageing. Plant Cell 20, 2909–2925. 10.1105/tpc.107.056341 18978034 PMC2590718

[B66] Schwacke R. Ponce-Soto G. Y. Krause K. Bolger A. M. Arsova B. Hallab A. (2019). MapMan4: a refined protein classification and annotation framework applicable to multi-omics data analysis. Mol. Plant 12, 897–892. 10.1016/j.molp.2019.01.003 30639314

[B67] Soubeyrand E. Colombié S. Beauvoit B. Dai Z. Cluzet S. Hilbert G. (2018). Constraint-based modeling highlights cell energy, redox status and α-ketoglutarate availability as metabolic drivers for anthocyanin accumulation in grape cells under nitrogen limitation. Front. Plant Sci. 9, 421. 10.3389/fpls.2018.00421 29868039 PMC5966944

[B68] Steinhauser M.-C. Steinhauser D. Koehl K. Carrari F. Gibon Y. Fernie A. R. (2010). Enzyme activity profiles during fruit development in tomato cultivars and *Solanum* pennellii. Plant Physiol. 153, 80–98. 10.1104/pp.110.154336 20335402 PMC2862428

[B69] Studart-Guimarães C. Gibon Y. Frankel N. Wood C. C. Zanor M. I. Fernie A. R. (2005). Identification and characterisation of the alpha and beta subunits of succinyl CoA ligase of tomato. Plant Mol. Biol. 59, 781–791. 10.1007/s11103-005-1004-1 16270230

[B70] Suda Y. Tachikawa H. Yokota A. Nakanishi H. Yamashita N. Miura Y. (2003). Saccharomyces cerevisiae QNS1 codes for NAD+ synthetase that is functionally conserved in mammals. Yeast 20, 995–1005. 10.1002/yea.1008 12898714

[B71] Turner W. L. Waller J. C. Vanderbeld B. Snedden W. A. (2004). Cloning and Characterization of two NAD kinases from *Arabidopsis*. Identification of a calmodulin binding isoform. Plant Physiol. 135, 1243–1255. 10.1104/pp.104.040428 15247403 PMC519044

[B72] Upmeier B. Thomzik J. E. Barz W. (1988). Nicotinic acid-N-glucoside in heterotrophic parsley cell suspension cultures. Phytochemistry 27, 3489–3493. 10.1016/0031-9422(88)80754-4

[B73] Waller J. C. Dhanoa P. K. Schumann U. Mullen R. T. Snedden W. A. (2010). Subcellular and tissue localization of NAD kinases from *Arabidopsis*: compartmentalization of *de novo* NADP biosynthesis. Planta 231, 305–317. 10.1007/s00425-009-1047-7 19921251

[B74] Wang G. Pichersky E. (2007). Nicotinamidase participates in the salvage pathway of NAD biosynthesis in *Arabidopsis* . Plant J. 49, 1020–1029. 10.1111/j.1365-313X.2006.03013.x 17335512

[B75] Wu R. Zhang F. Liu L. Li W. Pichersky E. Wang G. (2018). MeNA, controlled by reversible methylation of nicotinate, is an NAD precursor that undergoes long-distance transport in *Arabidopsis* . Mol. Plant 11, 1264–1277. 10.1016/j.molp.2018.07.003 30055263

[B76] Zhang X. Mou Z. (2009). Extracellular pyridine nucleotides induce PR gene expression and disease resistance in *Arabidopsis* . Plant J. 57, 302–312. 10.1111/j.1365-313X.2008.03687.x 18798871

