# Supplementary figures and images for: Regulation of Pyridine Nucleotide Metabolism During Tomato Fruit Development Through Transcript and Protein Profiling
Source: Front Plant Sci. 2019 Oct 11;10:1201. doi: 10.3389/fpls.2019.01201 (PMC6798084; doi:10.3389/fpls.2019.01201)

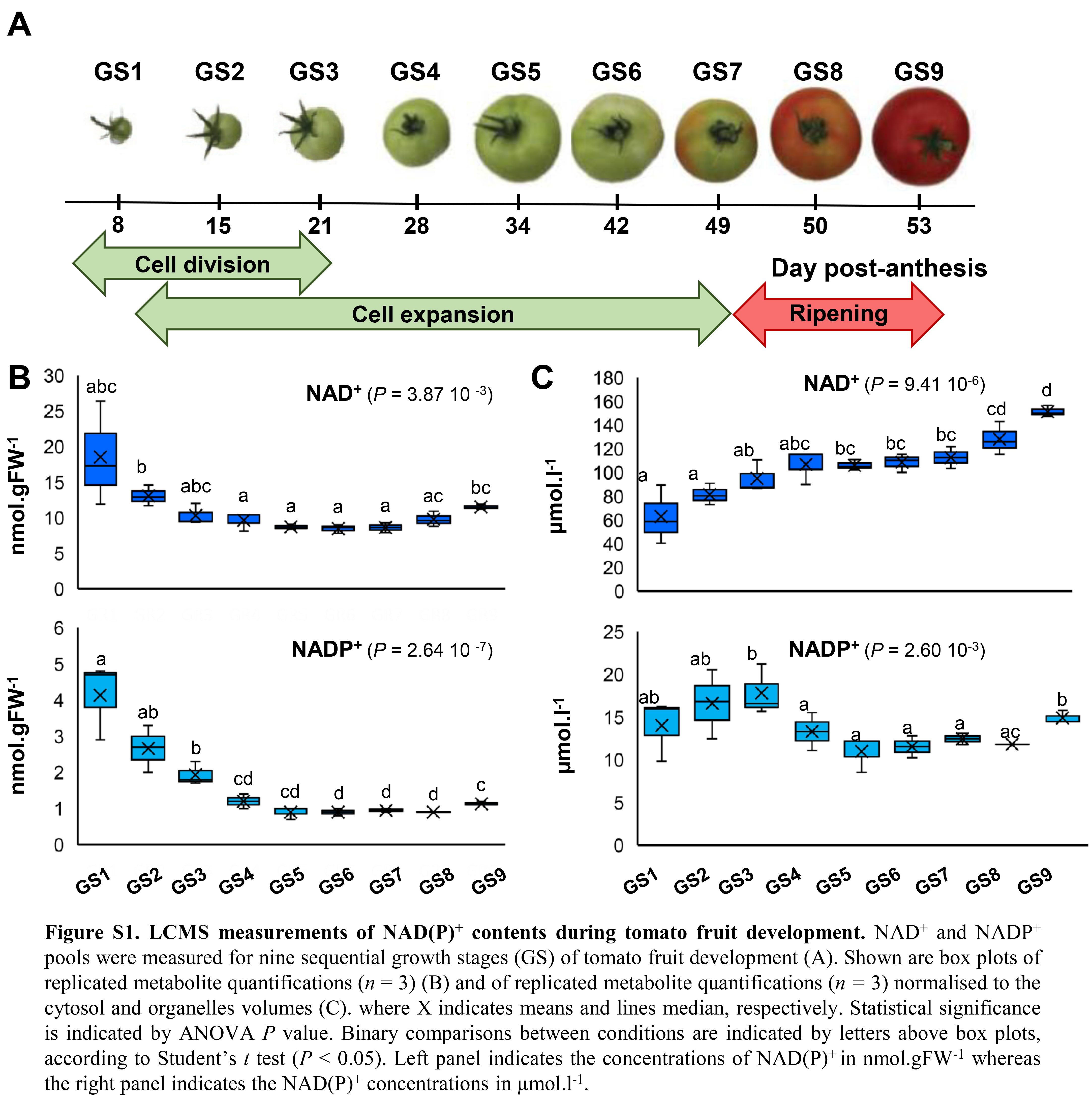

Supplement: Supplementary file 2 [file Image_1.tif]

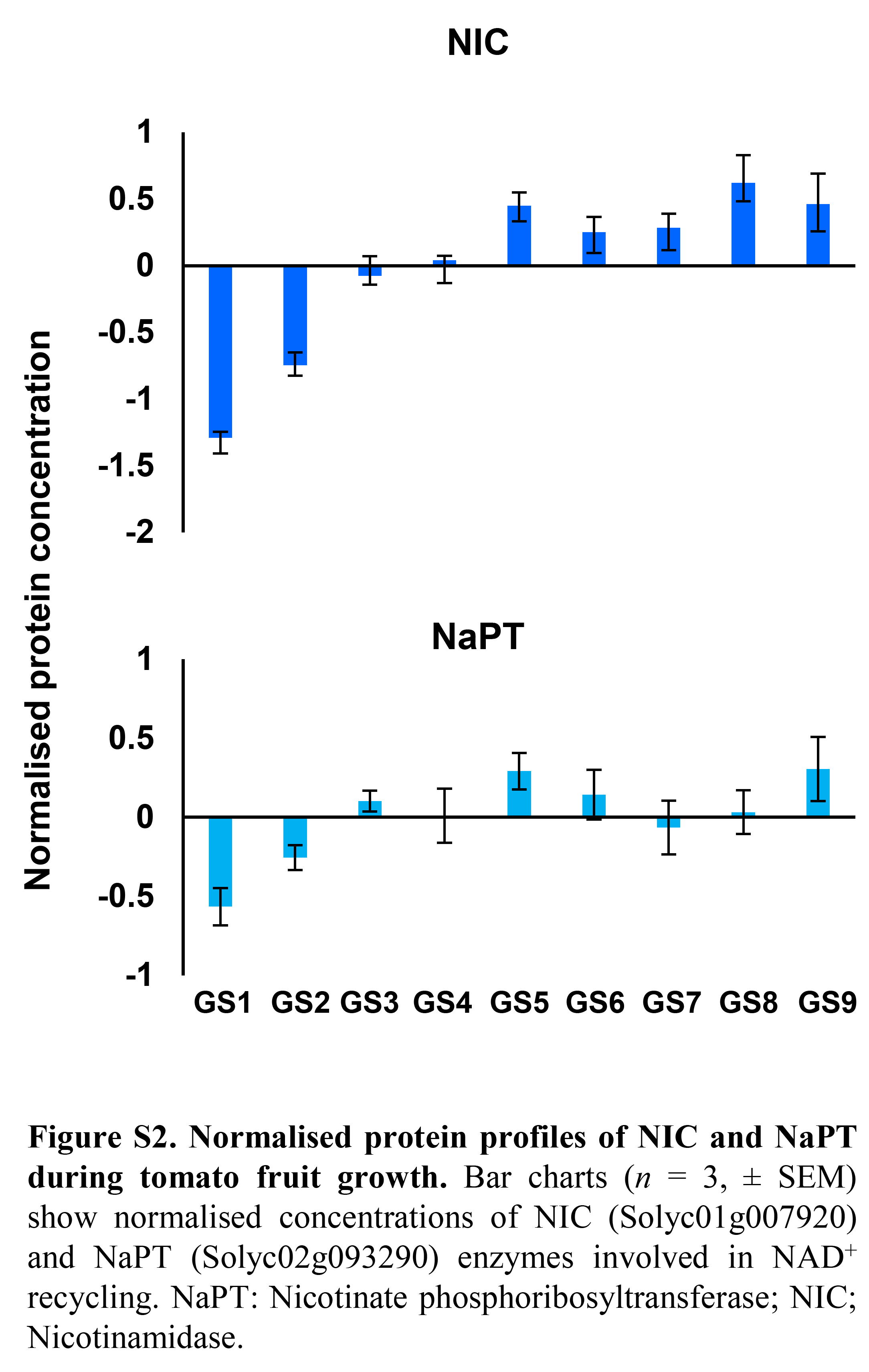

Supplement: Supplementary file 3 [file Image_2.tif]

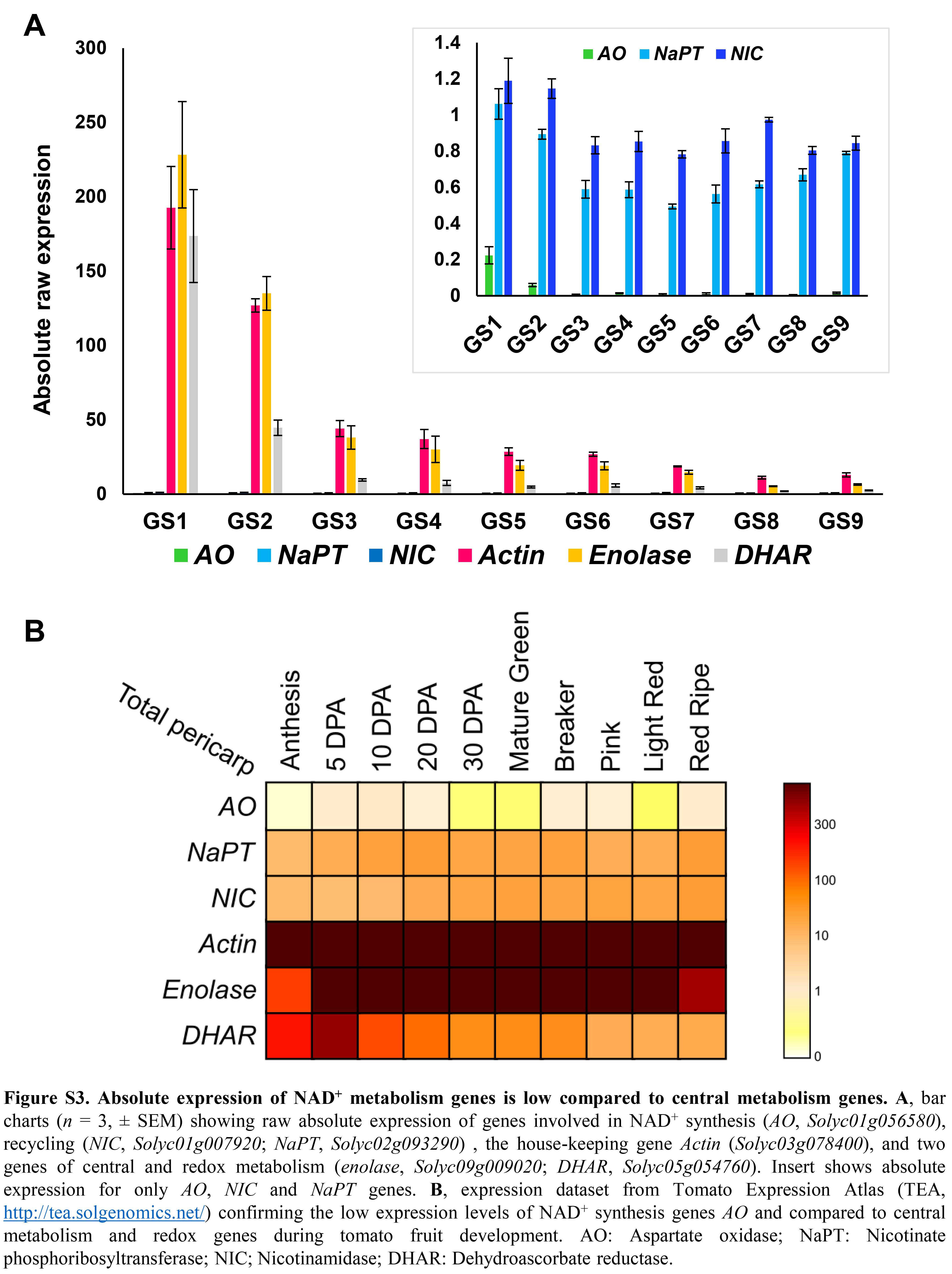

Supplement: Supplementary file 4 [file Image_3.tif]

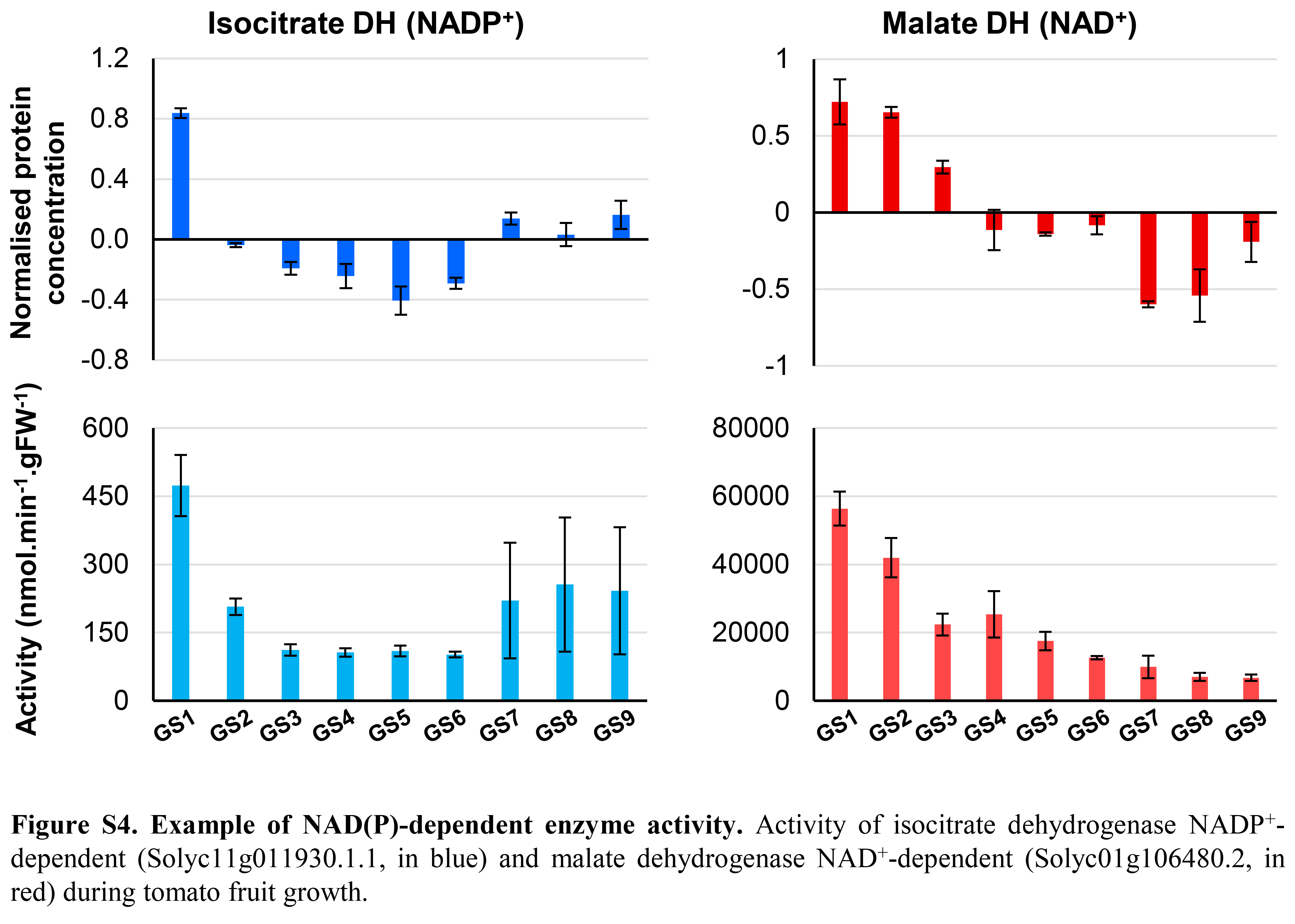

Supplement: Supplementary file 5 [file Image_4.tif]

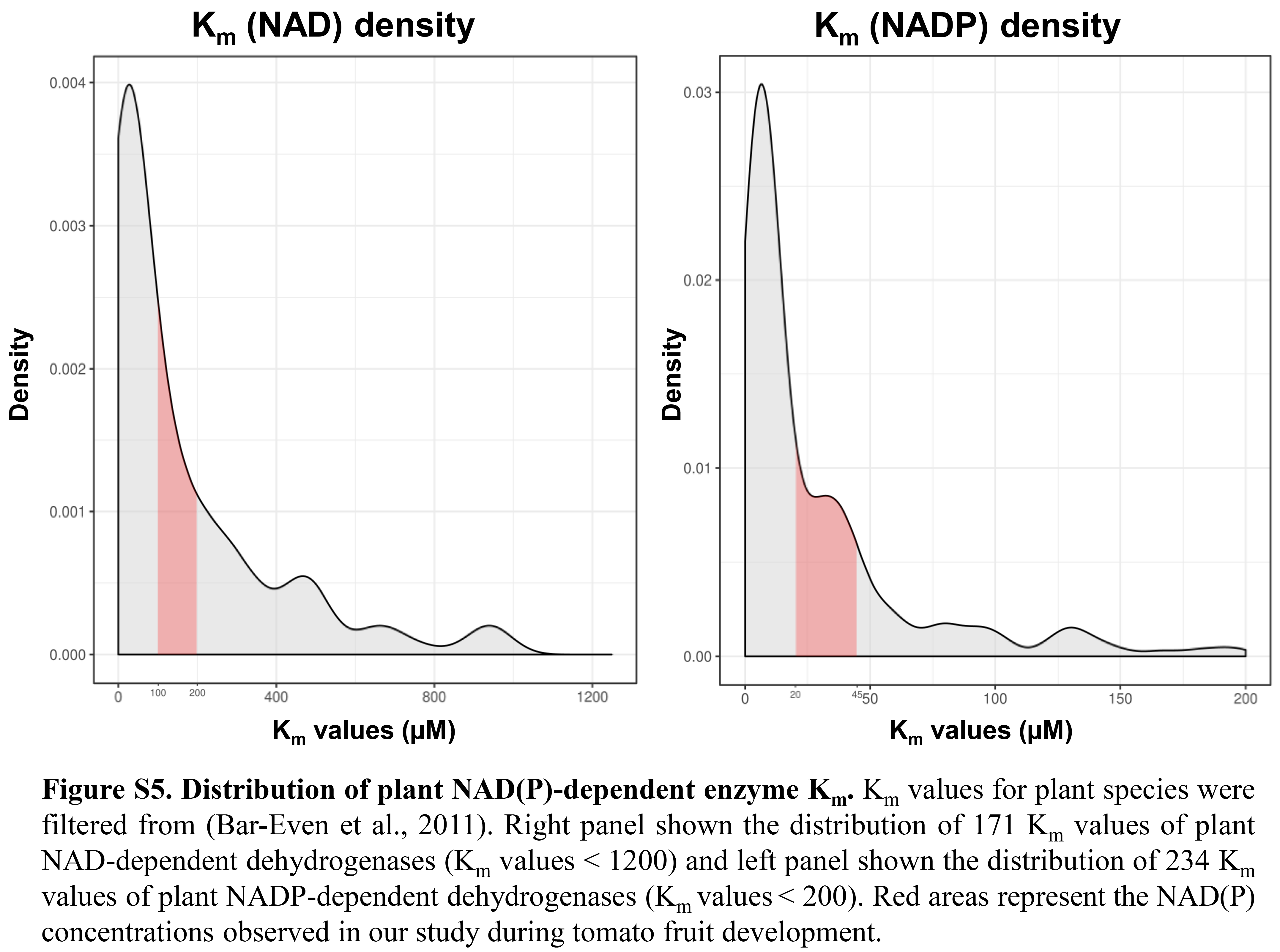

Supplement: Supplementary file 6 [file Image_5.tif]
